# Performance of Mixed Matrix Membranes Containing Porous Two-Dimensional (2D) and Three-Dimensional (3D) Fillers for CO_2_ Separation: A Review

**DOI:** 10.3390/membranes8030050

**Published:** 2018-07-28

**Authors:** Mahdi Ahmadi, Saravanan Janakiram, Zhongde Dai, Luca Ansaloni, Liyuan Deng

**Affiliations:** Department of Chemical Engineering, Norwegian University of Science and Technology (NTNU), NO-7491 Trondheim, Norway; mahdi.ahmadi@ntnu.no (M.A.); saravanan.janakiram@ntnu.no (S.J.); zhongde.dai@ntnu.no (Z.D.)

**Keywords:** mixed matrix membranes, CO_2_ separation, porous nanoparticles

## Abstract

Application of conventional polymeric membranes in CO_2_ separation processes are limited by the existing trade-off between permeability and selectivity represented by the renowned upper bound. Addition of porous nanofillers in polymeric membranes is a promising approach to transcend the upper bound, owing to their superior separation capabilities. Porous nanofillers entice increased attention over nonporous counterparts due to their inherent CO_2_ uptake capacities and secondary transport pathways when added to polymer matrices. Infinite possibilities of tuning the porous architecture of these nanofillers also facilitate simultaneous enhancement of permeability, selectivity and stability features of the membrane conveniently heading in the direction towards industrial realization. This review focuses on presenting a complete synopsis of inherent capacities of several porous nanofillers, like metal organic frameworks (MOFs), Zeolites, and porous organic frameworks (POFs) and the effects on their addition to polymeric membranes. Gas permeation performances of select hybrids with these three-dimensional (3D) fillers and porous nanosheets have been summarized and discussed with respect to each type. Consequently, the benefits and shortcomings of each class of materials have been outlined and future research directions concerning the hybrids with 3D fillers have been suggested.

## 1. Introduction

An wide scientific consensus is nowadays established in the international community over the anthropogenic climate change and global warming due to a drastic increase of atmospheric level of CO_2_ [1]. Anthropogenic activities within transportation, energy supply from fossil fuels [2], and raw materials (e.g., cement, steel) production [3] have significantly contributed to increase in levels of CO_2_ emissions over the last century, raising the CO_2_ concentration in the atmosphere [4]. The primary strategy to mitigate CO_2_ emission in the short term is carbon capture and sequestration (CCS), which mainly includes post-combustion (capture downstream to the combustion), oxy-fuel (purified O_2_ used for the combustion), and pre-combustion (capture upstream to the combustion) processes [2]. Furthermore, CO_2_ separation is relevant also for other applications, such as Natural Gas sweetening, where acid components in the presence of water can corrode pipelines and equipment, thus lowering the value of the natural gas [3,5]. Therefore, the development of efficient technologies to separate and capture CO_2_ is of primary interest.

Physical and chemical adsorption/absorption technologies have been widely applied to industrial plants to separate CO_2_ from gaseous streams. These conventional methods exploit pressure and temperature swing absorption/adsorption, which are typically energy-intensive and are not preferred from an environmental and economic standpoint [6]. The most mature technology for post combustion application is absorption using amine-base solvents, but, despite the efforts that are made, the increase in the cost of electricity would be still above the limit of 35%, which is identified as viable solution from a market perspective [7]. When compared to traditional technologies, membrane-based gas separation technology offers several advantages: lower energy consumption (no need for regeneration), no use of harmful chemicals, modularity and easier scalability. Additionally, membrane gas separation offers lower capital and operating costs. Depending on their base material, membranes used for CO_2_ separation can be separated in inorganic or polymeric. Even though inorganic membranes offer good separation abilities, polymeric materials are preferred for the application that requires large separation area, due to the lower production costs and easier processability. However, constant research is ongoing in order to improve the state-of-the-art separation for polymeric membranes, aiming at improving their competitiveness to traditional technologies.

Gas transport through a nonporous polymeric membrane is typically based on the “solution-diffusion” mechanism. Conceptually, the gas molecules is absorbed on the upstream side of the membrane layer, it diffuses across the thickness, and is finally desorbed on the downstream side. The permeation is therefore described as contribution of a thermodynamic parameter (solubility) and a kinetic factor (diffusivity), which affect the transport of gas molecules across the membrane matrix. The two most important features characterizing gas permeation membranes are permeability and selectivity [8]. Permeability of a given gaseous species (A) is as an intrinsic property of the material and is defined as the specific flux (*J_A_*) normalized on the membrane thickness (*ℓ*) and partial pressure difference between the upstream and downstream side of the membrane (Δ*p_A_*), as showed in Equation (1):

(1) $$\;P_{A}=\frac{J_{A\;}\cdot \;\ell }{\Delta p_{A}}\;$$

Permeability is frequently reported in Barrer (1 Barrer = 10^−10^ cm^3^ (STP) cm^−1^ s^−1^ cmHg^−1^ = 3.346 × 10^−16^ mol m^−1^ Pa^−1^ s^−1^). For the implementation of membranes in real process operations, membranenologists have to focus on the fabrication of thin composite membranes, aiming at maximizing the transmembrane flux of permeants [9]. In this perspective, the capacity of a membrane to allow for a specific gas to permeate through the selective layer is described by means of permeance, often reported in GPU (gas permeation unit, 1 GPU = 10^−6^ cm^3^ (STP) cm^−2^ s^−1^ cmHg^−1^ = 3.346 × 10^−10^ mol m^−2^ Pa^−1^ s^−1^). Unlike permeability, permeance is not an intrinsic property of the polymeric material, but it directly quantifies the actual transmembrane flux achievable for a given driving force. For this reason, the gas permeance is described as the ratio of the flux (*J_A_*) and the driving force (Δ*p_A_*). The other key membrane feature is the separation factor (or selectivity), which is defined as the molar ratio of gases *A* and *B* in the permeate (*y*) and in the feed side (*x*), with *A* being the most permeable gaseous species:

(2) $$\;\alpha =\frac{y_{A}/y_{B}}{x_{A}/x_{B}}\;$$

When single gas tests are performed, the membrane “ideal” selectivity can be estimated as the ratio between the permeability of the two penetrants [10].

The analysis of the performance of a larger amount of polymers for gas permeation allowed for Robeson [11,12] to highlight the existence of a trade-off between permeability and selectivity for materials governed by the solution-diffusion mechanism. This relation between permeability and selectivity reveals that for polymer membranes, an increase in permeability happens typically at the expense of selectivity, and vice versa. In the attempt to provide a more fundamental explanation, of an empirical relationship between permeability and selectivity was established [13,14], and it was shown that in the determination of the upper bound slope, the diffusion coefficient plays a dominant role as compared to the solubility coefficient. 

Among the different strategies to overcome the upper bound (fabrication of highly permeable polymers, such as thermally rearranged polymers [15], high free volume glassy polymers [16]; facilitated transport membranes [17]), a promising approach is the embedment of different phases (inorganic or liquid) within the membrane matrix, fabricating so-called hybrid membranes. Inorganic membranes that are made of non-polymeric materials, such as carbon molecular sieves, zeolites, or metal organic frameworks (MOFs) are typically characterized by performance exceeding the upper bound [18], but their cost and poor mechanical stability limit their applicability at large scale. Nevertheless, the dispersion of high performance nano-phases within a polymer matrix can significantly improve the neat polymer separation properties. In recent years, extensive efforts have been made in order to fabricated hybrid materials containing dispersed inorganic phases within polymeric matrices [8,19,20,21]. 

Based on the type of the embedded phase, hybrid membranes are classified in two main groups, known as mixed matrix membranes and nanocomposite membranes [10]. Nanocomposite membranes contain nano-sized impermeable nanoparticles that can contribute to the overall transport via surface adsorption or due to the presence of moieties with a specific affinity towards a specific penetrant. In our previous review, a broad overview of the performance of nanocomposite membranes has been presented [22]. On the opposite side, in mixed matrix membranes, the embedded phase contributes to a secondary transport mechanism. The fillers are typically porous and the pore architecture confers a larger CO_2_ solubility and/or diffusivity selectivity to the hybrid when compared to the neat polymer. Based on the nature of the embedded phase, the secondary transport mechanism can be described by molecular sieving, surface diffusion, or Knudsen diffusion. Nevertheless, the effect of the fillers on the overall transport through the hybrid membrane is inherently related to the type of polymer-particle interface that is achieved [10]. Ideal adhesion between the two phases would allow for achieving the largest enhancement, whereas poor interface morphology would result in the formation of unselective voids, frequently reflected by deteriorated separation performances.

We previously categorized [22] inorganic fillers in different categories based on their morphology (zero- to three-dimensional morphology), specifying which type constitutes the class of nanocomposite (zero-dimensional (0D) to two-dimensional (2D) nanofillers) or mixed matrix membranes (three-dimensional (3D) nanoparticles). Silica, metal oxide, nanotubes, nanofibers, and graphene derivate are categorized within the nanoparticles used for the fabrication of nanocomposite membranes, whereas zeolites, metal organic frameworks (MOFs), and porous organic frameworks (POFs) are listed as nano-phases that are used for the fabrication of mixed matrix membranes. 

The current report mainly focuses on the latest advances in hybrid membranes containing phases that are able to add secondary transport mechanisms of gas permeation in the polymer matrix, such as 3D nanofillers and porous nanosheets. Differently from other reviews recently reported [23,24,25,26,27], a systematical assessment of the impact of different porous nanomaterials on the CO_2_ separation performance of polymeric matrices is proposed, limiting the analysis mainly to the results reported in the last five years. The benefits that are related to the addition of the different porous nanofillers are discussed, categorizing the hybrid membranes according to the nature of the dispersed phases. The performances that are achieved by each dispersed phase are analyzed and compared among different polymeric matrices and loadings. This systematical analysis allows to identify the benefits and issues of each nanofiller type, offering an interesting tool to shape the direction of future research. The CO_2_ separation performance are analyzed for the gas pairs of interest for carbon capture (CO_2_ vs. N_2_ and CO_2_ vs. H_2_) and for natural gas and biogas purification (CO_2_ vs. CH_4_). If no numerical values were reported in the original manuscript to describe the performance, relevant information were carefully extracted via plots’ digitalization (WebPlotDigitizer, Version 4.1). 

## 2. Metal Organic Frameworks (MOFs)

MOFs represent a heterogeneous class of hybrid materials constructed from organic bridging ligands and inorganic metal nods [28]. When compared to traditional porous materials, such as zeolites, MOFs have drawn considerable attention thanks to their porous structure, large pore volume, fine tunable chemistry, and high surface area. MOFs are used in a large variety of applications, such as catalysis, sensing and electronic devices, drug delivery, energy storage, and gas separation [29,30,31]. In gas separation applications, recently, several efforts have been dedicated to the incorporation of MOFs in polymeric matrixes to produce hybrid membranes [20]. When compared to fully inorganic materials, such as Zeolites, the presence of organic ligands in the MOFs’ structure leads to better affinity and adhesion with polymers and organic materials [6], making MOFs extremely promising for the achievement of proper interface morphology, and thus, improved separation performance. Hydrothermal, solvothermal or sonication-assisted methods, microwave-assisted, and room temperature reaction are the synthesis procedures that are frequently reported for MOFs [32]. Surface porosity, pore volume, and particle size of MOFs can be finely tuned by controlling the effective synthesis parameters, such as temperature, concentration, time, and pH. Theoretically, the unlimited number of ligands and metal ions provide infinite MOFs combinations. 

MOFs frameworks can be either rigid or flexible. Rigid MOFs with tuned pore diameter could be a promising alternative to molecular sieves. The sieving behavior in rigid MOFs gives rise to considerably enhanced diffusion selectivity of gas pairs with different kinetic diameters, such as CO_2_/N_2_ or CO_2_/CH_4_. On the other hand, flexible structures undergo a considerable framework relaxation in the presence of external stimuli, such as host-gas interaction, pressure, temperature, or light [33,34,35]. Typically, this temporary structural transformability is a non-desirable effect, as it alters the initial sieving ability of the MOF structure [36]. The main structural rearrangements are typically referred as “gate opening” and “breathing” [33]. The former phenomenon is described as a transition from a closed and nonporous to a porous with open gates configuration upon the effect of external stimuli. As an example, ZIF-8 shows the swing in the imidazole linker and opening the narrow window at low to high pressure [37]. On the other side, the breathing effect is described as the abrupt expansion or compression of the unit cell. This is typically observed in MILs, where the structural transformation is referred as open pore, closed pore (cp), narrow pore (np), and large pore (lp) [34]. Linker rotation is another possible structural change, which is typically observed for UiO-66, where the benzene ring present on the organic ligand shows a rotational barrier that can be overcome at higher temperature [38,39]. Other important parameters that affect the transport properties of MOF nanoparticles are the pore volume and the surface area, as they mainly affect the gas sorption capacity of the MOF nanoparticles. In the case of CO_2_, for example, it has been reported that the presence of unsaturated open metal sites can greatly enhance the CO_2_ sorption capacity due to considerable polarizability and quadrupole moment. Open metal cations play as Lewis acidic nodes that strongly favors CO_2_ [40,41]. The occurrence of breathing is reported to significantly affect the pore volume, and, therefore, the gas sorption ability. For example, in the case of MIL-53, an expansion of the unit cell volume from 1012.8 Å^3^ to 1522.5 Å^3^ when the CO_2_ pressure is increased from 5 bar to 15 bar has been observed [36]. 

In the following sections, common MOFs that are used in fabricating mixed matrix membranes (MMMs) for CO_2_ separation have been grouped according to their type of metal ion constituting the MOFs’ architecture. Individual analyses of gas permeation have been dedicated to the MMMs containing Zeolitic Imidazolate Frameworks (translational metal ions), UiO-66 (Zr-based), CO_2_-philic MOFs (Cu-based) and Materials Institute Lavoisier MOFs (trivalent metal ions). Other new and emerging MOFs have also been listed together in a separate section.

### 2.1. Zeolitic Imidazolate Frameworks (ZIFs)

**Figure 1:**
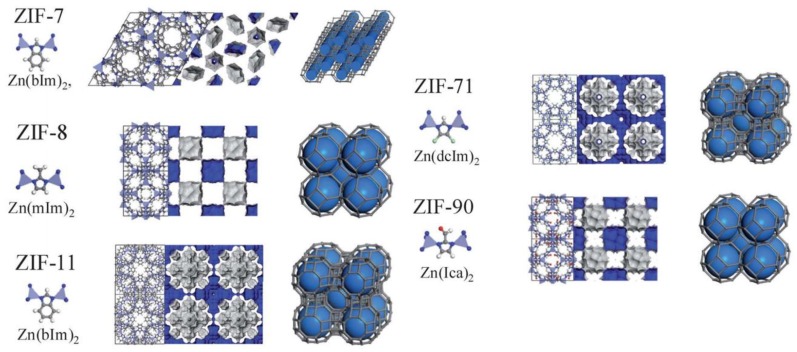
Zeolitic Imidazolate Frameworks (ZIF) structures with building blocks, topology, and accessible surface area for a probe diameter of 2 Å. Adapted from [42], with copyright permission from © 2012, Royal Society of Chemistry.

Zeolitic imidazolate frameworks, known as ZIFs, have received great attention due to their exceptional transport properties [42]. Generally, ZIFs are a subclass of metal organic frameworks with a zeolite, like topology, consisting of large cavities linked by narrow apertures [1]. ZIFs are composed of M-Im-M, where M stands for transitional metal ions (such as Zn, Cr) and Im is the organic linker (imidazolate and its derivatives), respectively. M-Im-M forms a 145° angle, which is similar to Si-O-Si angle in conventional aluminosilicate zeolites and makes structures analogous to zeolites with topologies of *sod*, *rho*, *gme*, *lta,* and *ana* [30,43]. Among the different ZIFs that are available [42], ZIF-7, ZIF-8, ZIF-11, ZIF-71, and ZIF-90 (Figure 1) are the most common MOFs incorporated in polymer matrix to produce hybrid membranes for carbon capture applications. 

#### 2.1.1. ZIF-8

ZIF-8 with *sod*-type topology and tetrahedral structure is the most frequently investigated MOF among the ZIFs family, which exhibits good thermal and exceptional chemical stability [44,45]. ZIF-8 has large pores of 11.8 Å and the pore limiting diameter of 3.4 Å, which represents a perfect sieving range for gas separation, such as CO_2_/N_2_ and CO_2_/CH_4_ [43]. However, the ZIF-8 framework is rather flexible, owing to the swing effect of organic linker that significantly affects the sieving ability [37,46]. This swing effect, which is supported experimentally and theoretically, was described by the rotation of imidazolate linker oscillating between two configurations of open window and close window [47]. The separation properties of ZIFs have been examined and researchers have explored their potential in the use of composite membranes for gas separation.

Matrimid^®^ is a commercial glassy polyimide, which is widely used as polymer basis for comparison of MOFs’ separation performance. Ordonez et al. [48] fabricated ZIF-8/Matrimid^®^ mixed matrix membranes with nanoparticles loading up to 80 wt.% and investigated their transport properties for CO_2_/N_2_ and CO_2_/CH_4_ separation at 2.6 bar and 35 °C. ZIF-8 with a size range within 50–150 nm were dispersed in chloroform together with the polymer and self-standing membranes were obtained via solvent casting and dried at 240 °C under vacuum. While increasing the ZIF-8 loading, the tensile strength of the hybrid matrix dropped significantly and samples with 80 wt.% loading were found too brittle to be tested. Interestingly, the analysis of the transport properties showed a double behavior of the hybrids. Up to 40 wt.%, the disruption of the chain packing that is produced by the presence of the nanoparticles resulted in an increase in free volume, and consequently, in gas permeability. A 158% increase in CO_2_ permeability (Table 1) was observed, even though the variation took place independent from the gas nature. On the contrary, at 50 and 60 wt.% loading the gas permeability dropped significantly, showing a considerable increase in the selective feature (CO_2_/CH_4_). The authors suggested a transition from a polymer-based to a ZIF-8-regulated transport, with the sieving effect of the fillers becoming dominant above a certain inorganic content. Interestingly, despite the CO_2_-philic nature of ZIF-8, the hybrid samples maintained the H_2_-selective features of the neat polymer (Table 1), but the low selectivity values (H_2_/CO_2_ < 5) are not of interest for the industrial applications. The CO_2_ separation performances of ZIF-8/Matrimid hybrid membranes have also been investigated by Basu et al. [49], limiting the loading up to 30 wt.%. SEM imaging showed the formation of a proper interface morphology between the particles and the polymer phase. Similar to the previous case, the CO_2_ permeability increased proportionally to the loading, reaching a 209% enhancement when compared to the neat polymer at the maximum loading. Possibly, the larger enhancement compared to the previous case may be attributed to the larger ZIF-8 size (250–500 nm). However, the separation factor appeared to be hardly affected by the presence of nanoparticles, with a maximum enhancement of 15%. Interestingly, the authors also compared the performance of other two MOFs (MIL-53 and Cu_3_(BTC)_2_), observing that the enhancement in CO_2_ permeability is mainly dependent on the loading, whereas the nanoparticles nature and size play a minor role in affecting the transport properties. Song et al. [50] synthesized ZIF-8 with particle size of about 60 nm, and fabricated mixed matrix membranes by embedding them into Matrimid. Morphological analysis showed a proper polymer/particle interface up to the maximum loading investigated (30 wt.%). Notably, the smaller ZIF-8 size determined a 250% enhancement in CO_2_ permeability at the highest loading, even though a negative effect on selectivity was observed (25% decrease at 30 wt.% loading) for both CO_2_/N_2_ and CO_2_/CH_4_.

**Figure 2:**
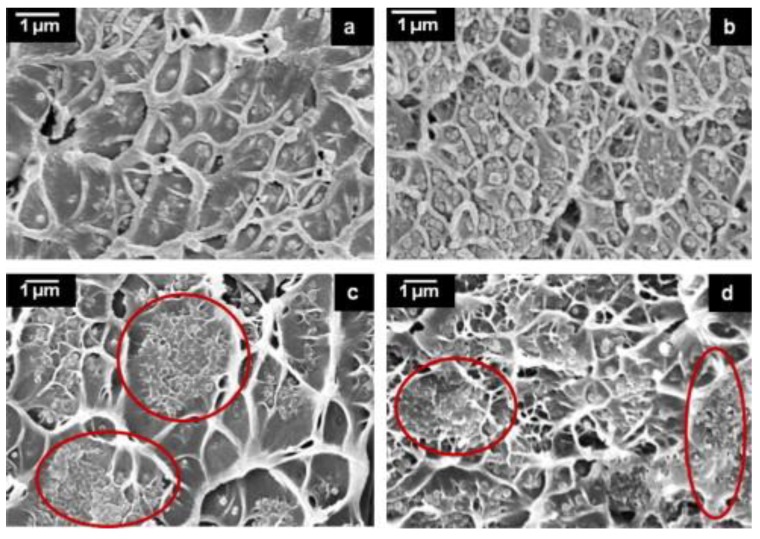
Dispersion of ZIF-8 by direct (**a**,**b**) and indirect (**c**,**d**) sonication of 10 wt.% (**a**,**c**) and 25 wt.% (**b**,**d**) loading in Matrimid [51], with copyright permission from © 2012 Elsevier.

Sonication has also been reported to be an important factor affecting the performance of ZIF-8-based mixed matrix membranes [51]. ZIF-8 nanoparticles were dispersed into Matrimid, exposing the casting solution to direct (sonication horn) or indirect (sonication bath) ultrasound wave (Figure 2). The study showed that different sonication intensities produced a significant change in the morphology of the nanoparticles, with limited influence on crystallinity and microporosity. When higher sonication intensity was applied to the casting solution, a proper interfacial morphology was achieved, with a simultaneous increase of permeability and selectivity (Table 1) and full consistency with the Maxwell model. When indirect sonication was employed, nanoparticles agglomeration was observed, affecting the efficiency of the hybrid membranes. ZIF-8 modification using mixed organic ligand (2-aminobenzimidazole as a substitution linker) has also been reported [52], leading to differences in pore size distribution and porosity when compared to pristine ZIF-8. When hybrid membranes were prepared while using Matrimid as polymer phase, no gate opening effect or structural flexibility was observed, and the ideal selectivity improved (Table 1). An interesting approach to improve the interface morphology has been proposed by Casado Coterillo et al. [53], who fabricated a ternary system, embedding ZIF-8 in a polymer matrix composed of Chitosan and [Emim][Ac]. At low ZIF-8 loading (5 wt.%), they achieved the best CO_2_/N_2_ separation performance and attributed the effect to a better adhesion between the Chitosan and the ZIF-8 phase that is offered by the presence of the ionic liquid at the interface. 

**Figure 3:**
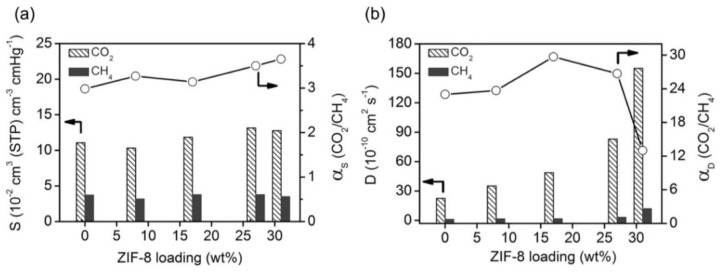
Effect of ZIF-8 loading on the solubility and the diffusivity selectivity when embedded in P84 polyimide [55], with copyright permission from © 2018 Elsevier.

Carter et al. [54] loaded 10% ZIF-8 with particle size of 95 nm in Matrimid and prepared two different dense membrane films with aggregated ZIF-8 nanoparticles and with a homogeneous dispersion. As expected, the single gas permeation tests showed improved selectivity and permeability for the well-dispersed membrane and the lower drop observed for the N_2_ permeability, with respect to CH_4_ permeability, was explained in terms of surface diffusion mechanism and framework flexibility of ZIF-8. Again, the addition of ZIF-8 nanoparticles enhanced the H_2_-selective properties of the hybrids, with the aggregated samples showing even better performance (68% increase in H_2_ permeability) when compared to the one with homogeneous dispersion (Table 1). However, the selectivity remained too low (H_2_/CO_2_ < 5) to become valuable for real H_2_ purification. Interestingly, the reported analysis of hybrid membranes based on Matrimid and ZIF-8 clearly showed that synthesis protocol, particle size, and possible modification play a major role in the determination of the membrane performance. Guo et al. [55] recently investigated the effect of ZIF-8 nanoparticles on another commercial polyimide, P84. As reported for Matrimid, the CO_2_ permeability increased proportionally to the MOF content. Also, the CO_2_/CH_4_ selectivity increased remarkably, but at the highest loading (31 wt.%), a drop (Table 1) was observed. A drop in the diffusion selectivity was measured (Figure 3), clearly suggesting that the formation of interfacial voids that are associated to MOFs aggregation is responsible for the observed phenomenon.

6FDA is another glassy polyimide that has been largely investigated for the fabrication of ZIF-based mixed matrix membranes. The higher free volume when compared to Matrimid allows for the 6FDA polymer family to achieve larger gas permeation, offering a more suitable option for industrial applications. Jusoh et al. [56] reported significant improvement in CO_2_ permeability of 6FDA-durene by embedding up to 20 wt.% ZIF-8 in the polymer matrix. An optimum loading of 10 wt.% was identified (Table 1), as a further increase of the inorganic content led to negligible enhancement of CO_2_ permeability, but a significant decrease of CO_2_/CH_4_ selectivity. Furthermore, the gas separation enhancement of ZIF-8/6FDA-durene was attributed to the influence of pore limiting diameter and quadrupole interaction of CO_2_ with the ligand in ZIF-8 framework. Wijenayake et al. [57] proposed surface crosslinking as possible approach to improve the performance of 6FDA-based hybrid membranes containing ZIF-8 nanoparticles. The addition of 33 wt.% ZIF-8 in the polymer matrix enhanced significantly the CO_2_ permeability (~400%, Table 1), reaching up to ~1500 Barrer, similar to the one that was observed in the previous study. The effect on the selectivity was limited. Even though post-synthetic modification of ZIF-8 using ethylenediamine showed enhanced CO_2_ adsorption capacity [58], the use of ethylenediamine vapors to crosslink the surface of the hybrid membrane led to a limited improvement on the CO_2_ selectivity along with a drastic drop in CO_2_ permeability. As in the case of Matrimid, the addition of ZIF-8 to 6FDA polyimide improved the H_2_-selective feature, and a H_2_/CO_2_ selectivity of 12 has been achieved upon surface modification. Askari and Chung [59] studied the effect of annealing temperature on the performance of 20 wt.% ZIF-8 containing 6FDA-durene mixed matrix membrane by heating to different temperature (200, 350, and 400 °C) below glass transition temperature (T_g_ > 400 °C). The highest gas permeability was obtained for 20 wt.% loaded membrane annealed at 400 °C (from 487 Barrer at 200 °C to 1090 Barrer at 400 °C) and the contribution of the inorganic phase was enhanced at higher annealing temperatures. When the cross-linkable co-polyimide (6FDA-durene/DABA) was used in the place of the homopolymer, higher selectivity values could be achieved, but the improvement took place to the detriment of CO_2_ permeability. Nafisi and Hägg investigated the gas separation performance of ZIF-8 containing membrane prepared using 6FDA-durene [60] and PEBAX 2533 [61] (a commercial polyether-block-amide) as polymer phase. In both cases, the CO_2_ permeability increased along with the inorganic content, but the influence of ZIF-8 nanoparticles appeared to be more effective for PEBAX 2533. At 30 wt.% loading, a 50% enhancement of CO_2_ permeability (2186 Barrer) was observed for 6FDA-durene whereas a ZIF-8 loading of 35 wt.% in PEBAX 2533 corresponded to a 3.6-fold improvement of the CO_2_ permeability (1287 Barrer). Furthermore, at high inorganic loading, the polyimide showed reduced CO_2_ selectivity, whereas negligible effect on the separation performance was observed for PEBAX. 

Recently, Sanchez-Lainez et al. [62] reported the fabrication of mixed matrix membranes based on polybenzimidazole (PBI), obtained via phase inversion method for H_2_/CO_2_ separation. At 180 °C, the presence of the ZIF-8 nanoparticles improved the H_2_/CO_2_ selectivity as well as the H_2_ permeance. At higher temperature (250 °C), the presence of defects resulted in a drop in the selective characteristic, but higher feed pressure (3 bar vs 6 bar) restored the H_2_/CO_2_ selectivity to a value close to 20.

Recent publications showed an increasing research also on the fabrication of thin composite membranes containing ZIF-8 nanoparticles. Dai et al. [63] fabricated asymmetric hollow fiber mixed matrix membranes using dry jet-wet quench method. In particular, they dispersed 13 wt.% ZIF-8 nanoparticles (size ~200 nm) into a polyetherimide (Ultem 1000) matrix. CO_2_/N_2_ separation performance for the HF membranes were tested at 35 °C and 100 psi. For both pure and mixed gas, the separation performance was improved. The permeance and selectivity of the ZIF-8 containing hollow fibers improved by 85% and 20%, respectively, when compared to the unloaded hollow fibers. Higher selective feature were observed for mixed gas conditions using 20 vol.% CO_2_ in the feed. A comprehensive review on progresses and trends on hollow fiber mixed matrix membranes has been recently reported by Mubashir et al. [64]. The review includes a comparison between the results obtained for flat sheet and hollow fiber mixed matrix membranes at similar filler loading and operating conditions. It was concluded that hollow fiber mixed matrix membranes that are loaded with ZIF-8, ZIF-93, and amine functionalized MILs show higher separation performance for CO_2_/N_2_ and CO_2_/CH_4_.

Thin film can be obtained also by coating on porous support. Thin film composite membranes and thin film nanocomposite membrane containing MOFs have been developed for nanofiltration and organic solvent separation [65,66,67,68]. However, only few studies can be found in literature investigating the gas transport properties of thin hybrid selective layers. Sánchez-Laínez et al. [69] reported a novel ultra-permeable thin film nanocomposite (TFN) containing ZIF-8 for H_2_/CO_2_ separation. The selective layer (50–100 nm) was formed on a polyimide P84 asymmetric support. The nanoparticles were dispersed in different loadings (0.2, 0.4, and 0.8% *w*/*v*) in a polyamide matrix. The incorporation of ZIF-8 nanoparticles enhanced the gas separation performance. At 35 °C and 0.4% *w*/*v* ZIF content, a 3-fold increase in selectivity was observed compared to the pristine polymer. An increase in the temperature had a positive impact on the performance, especially in terms of H_2_ permeance (up to 988 GPU at 250 °C for the pristine polymer). At 180 °C, TFN membranes containing 0.2 and 0.4% (*w*/*v*) of ZIF-8 exhibited a marked selectivity increase of 42% and 64%, respectively. At higher loading (0.8% *w*/*v*), the presence of micro voids and defects determined a significant drop in both permeance and selectivity. A further increase in temperature led to higher H_2_ permeance of TFN membranes with negligible influence on the selective features.

#### 2.1.2. ZIF-7

ZIF-7 is another promising candidate of the ZIFs family for gas separation applications. 1H-benzimidazole is the bridging ligand, which is connected to the Zn metal clusters and creates a 3D sodalite topological framework (Figure 2). Its pore diameter ranges between 3 and 4.3 Å [44,70]. The narrow pore size makes ZIF-7 suitable for H_2_ purification from CO_2_. Nevertheless, due to the flexibility of the benzimidazole linker, ZIF-7 also shows the “gate opening effect”, undergoing a reversible transition of the pores (from narrow to large framework flexibility of ZIF-7 that allows for gas molecules with a molecular diameter as large as 5.2 Å to access the pores and cavities). This gate opening effect of ZIF-7 was observed in adsorption isotherms (CO_2_, ethane, and ethylene) [71]. 

Li et al. [72] evaluated the separation performance of ultrathin hybrid membrane composed by a poly(amide-b-ethylene oxide) (Pebax 1657) and ZIF-7 nanoparticles. ZIF-7 particles with a size between 40 and 50 nm were synthesized and embedded up to 34 wt.% within the polymer matrix. Subsequently, thin composite membranes were prepared by coating the casting solution on a porous PAN support (PTMSP gutter layer was used to prevent pore penetration of the selective layer). Increasing the ZIF-7 loading up to 22 wt.% showed a remarkable increase (Table 1) in both CO_2_ permeability and CO_2_/CH_4_ and CO_2_/N_2_ ideal selectivity. However, at higher loading (34 wt.%) polymer rigidification around the nanoparticles took place, positively affecting the selectivity (214% and 208% enhancement for CO_2_/CH_4_ and CO_2_/N_2_, respectively), while the CO_2_ permeability was considerably lower when compared to that of the neat polymer. Post synthesis modification of nanosized (40–70 nm) ZIF-7 was implemented by Al-Maythalony et al. [73], aiming at tuning the pore size by exchanging the organic ligand, benzimidazolate with benzotriazolate. The synthesized nZIF-7 and PSM-nZIF-7 were embedded in a polyetherimide (PEI) matrix. The post synthesis modification resulted in an increase of CO_2_ permeability of all the examined gases (N_2_, CH_4_, and CO_2_ by 737%, 470%, and 198%, respectively). Nevertheless, the bigger enhancement of gases with larger kinetic diameters reduced the CO_2_-selective feature of the hybrids when compared to the pristine PEI. 

#### 2.1.3. ZIF-11, ZIF-71, and ZIF-90

ZIF-11, ZIF-71, and ZIF-90 are the other three structures from the ZIFs library that are of interest for gas separation applications and are characterized by *rho* (for both ZIF-11 and ZIF-71), and *sod* type topology with apertures of 3 Å, 4.2 Å, and 3.5 Å, respectively [45,74]. ZIF-90 is an attractive MOF for CO_2_ capture owing to its covalent carbonyl bond in the imidazole linker favoring CO_2_ and the 0.35 nm of pore size, which is suitable for CO_2_/CH_4_ separation. Alternatively, ZIF-71 is selected due to its large cavity pore diameter (16.5 Å) when compared to that of ZIF-8, ZIF-90, and ZIF-11 (cavity pore diameter 11.6, 11.2, and 14.6 Å, respectively) that has the potential to enhance the gas separation performance of hybrid membranes [75,76]. 

Ehsani and Pakizeh [77] examined the performance of hybrid membranes with a ZIF-11 loading range of 10–70 wt.% incorporated into PEBAX 2533. Morphological characterization of MMMs revealed an excellent adhesion between the polymer matrix and the nanoparticles. Even at 50 to 70 wt.% ZIF-11 loading, no significant agglomeration could be observed, even though poorer interfacial morphology appeared. At lower MOF loading, the presence of polymer chain rigidification and pore blockage resulted in a gas permeability reduction (~20%). At higher loading (>50 wt.%), the CO_2_ permeability increased when compared to pristine polymeric membrane, reaching a value of 403 Barrer at 70 wt.% (Table 1). Different effects were observed for selectivity: the CO_2_/CH_4_ selectivity increased from 8 to 12.5 at increasing the MOF content, but a negative trend was observed in the case of CO_2_/N_2_ selectivity. ZIF-11 has also been embedded in 6FDA-DAM polyimide [78]. SEM micrographs showed no apparent agglomeration for loading up to 30 wt.%. An optimum was observed incorporating 20 wt.% ZIF-11, leading to a 12-fold enhancement of CO_2_ permeability (Table 1), with limited effect on the ideal selectivity. The CO_2_ permeability improvement was associated to the achievement of particles alignment, and subsequently, an increase in fractional free volume of the hybrid matrix, which is confirmed by d-spacing analysis. The lack of selectivity improvement for 20 wt.% loading was related to the much higher gas permeability of ZIF-11 as compared to 6FDA-DAM, as predicted by the Maxwell model. Further increase in ZIF-11 loading did not show any improvement of the separation performance, owing to polymer chain rigidification and pore blockage. 

**Figure 4:**
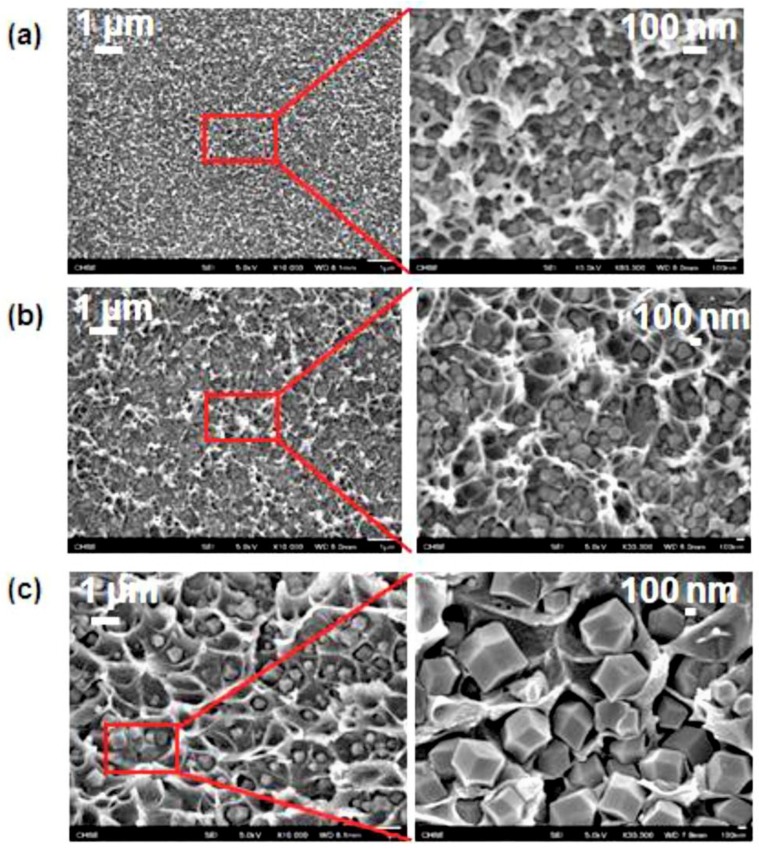
Cross-sectional morphology of 6FDA-Durene containing ZIF71 particles with average size of 30 nm (**a**); 200 nm (**b**); and 600 nm (**c**) [76], with copyright permission from © 2016, American Chemical Society.

Hybrid membranes based on PIM-1 and ZIF-71 with various loading were fabricated by Hao et al. [79]. The addition of ZIF-71 into PIM-1 considerably improved the gas transport, and in the case of CO_2_, the permeability value increased from 3295 to 8377 Barrer (Table 1). Photo oxidation obtained via UV treatment of the neat polymeric matrix increased the ideal selectivity to the detriment of gas permeability. As expected, the presence of the nanofillers helped in minimizing the gas permeability drop, showing impressive membrane performance (CO_2_ permeability of 3459 Barrer, CO_2_/CH_4_ and CO_2_/N_2_ selectivity of 35.6 and 26.9, respectively) [79]. The effect of particle size (30, 200, and 600 nm, as seen in Figure 4) has also been investigated, using a fixed amount of nanoparticles in 6FDA-durene (Table 1) [76]. The permeability enhancement associated to the presence of the nanoparticles did not scale with the particle size, but it showed an optimum when the 200 nm particles size were used. In addition, the negligible effect on the ideal gas selectivity suggested the existence of a trade-off between the particle size and the gas separation performance, giving an important indication for the further development of nano-hybrid membranes.

Bae et al. [80] studied the CO_2_ separation performance of MMMs containing a fixed amount of ZIF-90 (15 wt.%), coupled with three different polyimides (6FDA-DAM, Matrimid and Ultem), aiming at determining the effect of the nanofillers on different polymer phases. In the case of Matrimid and Ultem, the CO_2_ permeability increased (~100%, Table 1). As previously reported, the negligible selectivity variation observed is related to the higher gas permeability of the nanoparticles, as predicted by the Maxwell model. When a more permeable matrix was used (6FDA-DAM), the CO_2_ permeability improvement was followed by an increase of the CO_2_-selective features of the hybrid matrix. Mixed gas permeation tests showed separation performances well above the CO_2_/CH_4_ and CO_2_/N_2_ upper bounds. Zhang et al. [81] utilized ZIF-90 as the filler in triptycene-based polymer and prepared hybrid membranes for CO_2_/N_2_ and CO_2_/CH_4_ separation. Cross-sectional SEM images revealed homogenous dispersion of the nanofillers and membranes with defect-free interfacial morphology, even at high loadings. The membrane containing 50 wt.% ZIF-90 showed a 215% increase of the CO_2_ permeability value (Table 1), without sacrificing the gas selectivity. The ability of ZIF-90 to disrupt the polymer chain packing, and consequently, increase in free volume, was also suggested as source of additional permeability enhancement. 

According to the analysis of different ZIFs in different polymeric materials, it appears that it is possible to achieve relatively high loading of isotropic ZIFs particles in the polymer matrix (up to 60 wt.%). However, the optimum concentration of inorganic nanofillers appeared to be in the range of 30 to 40 wt.%; at higher loading, no significant benefits for CO_2_ permeability can be obtained, but a decrease in selectivity can be expected. The use of ZIFs has been demonstrated to also be successful for highly permeable polymer (6FDA-based polymides, PIM-1, PEBAX), and typically the introduction of nanoparticles has the main function of disrupting the polymer chain packing and increasing the free volume in the hybrid matrix. However, despite the achievement of suitable interface morphology, the addition of ZIFs to polymer matrix seldom is reported to have a significant impact on the selective feature of the mixed matrix membrane. Among the investigated ZIFs, it is not possible to identify one type that is able to stand out, but the efficiency of each type also depends on the chosen polymeric phase and the synthetic procedures. Furthermore, ZIF nanoparticles with smaller size appears to be more effective when compared to inorganic phases with bigger average size. Finally, despite the CO_2_-philic nature of the nanofillers, the incorporation of ZIFs in polymeric matrix typically enhances the H_2_-selective feature of the pristine polymeric matrix. 

### 2.2. Zirconium 1,4-Dicarboxybenzene (UiO-66)

**Figure 5:**
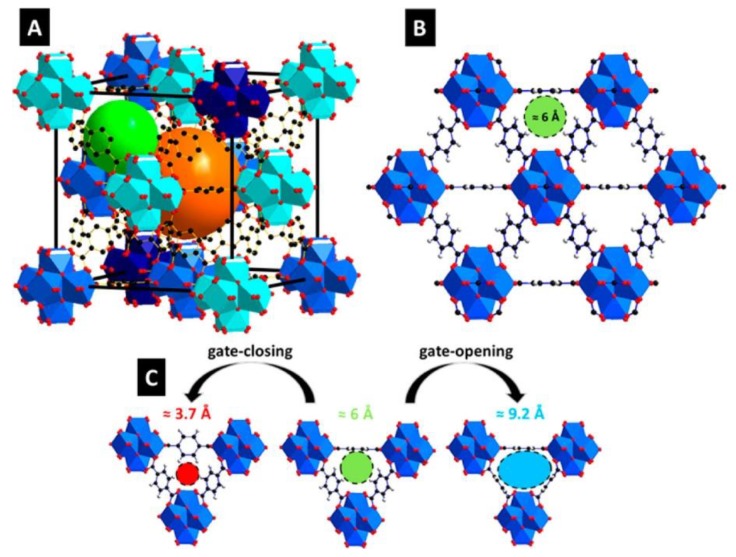
Three-dimensional (3D) structure of UiO-66 (**A**) visualizing the octahedral cage (orange) and the tetrahedral cage (green). Triangular windows (**B**) between the octahedral and tetrahedral cages. Pore opening changes upon rotation of the benzene ligands (**C**) [84], with copyright permission from © 2017, American Chemical Society.

UiO-66 is a zirconium-based metal-organic framework that is built from zirconium oxide (Zr_6_O_4_(OH)_4_) nodes linked together by 1,4-benzendicarboxylate as a bridging ligand [82]. UiO-66 is the first member of zirconium based MOFs family with *fcu*-topology introduced by Cavka et al. [83]. It owns a Langmuir surface area of 1187 m^2^/g and the narrow triangular windows that are present in the UiO-66 framework have different sizes (Figure 5): 6 Å is the window connected to the two octahedral cages, with the size of 11 Å, and the tetrahedral cage, which has an opening of 8 Å (Figure 5). UiO-66 showed exceptional mechanical and chemical stability on exposure to high temperature, up to 500 °C, and chemicals, making this MOF a promising candidate for many applications [82,84]. The benzene ring has been found to be characterized by the rotational barrier as compared to other MOFs, leading to changes in the pore opening size (Figure 5C), and this effect showed a temperature dependency behavior [85].

Hybrid membranes embedding 5 to 20 wt.% pristine UiO-66 and amine functionalized UiO-66-NH_2_ (average size 60–80 nm) in PEBAX 1657 have been prepared [86]. For both types of nanoparticles, the CO_2_ permeability increased proportionally to the amount of inorganic phase, reaching a ~2.5-fold enhancement of the pristine polymer value (Table 2). These results suggested that UiO-66 showed a strong affinity towards CO_2_ due to the presence of OH coordinated bond connected to Zr cluster. Different trends were observed for the CO_2_/N_2_ selectivity, which showed an optimum between 7.5 and 10 wt.% loading. The better affinity of the UiO-66-NH_2_ with the polymer phase allowed for reaching better selectivity improvement (88%) as compared to the pristine MOF (42%). Interestingly, the mixed matrix membranes prepared with UiO-66-NH_2_, retained stable performances even in the presence of humidity. Similar nanoparticles (UiO-66 and UiO-66-NH_2_) have been embedded also into Matrimid 9725 [87]. The use of two modulators (benzoic acid, BA, and 4-aminobenzoic acid, ABA) was reported to allow for the linkage of the amine groups in different positions in the UiO-66 structure. The presence of the ABA modulator increased the CO_2_/CH_4_ selectivity up to 55% (from 31.2 to 47.4), together with a six-folds improvement of the CO_2_ permeability for the amine-modified UiO-66 (Table 2). Surface modification of the UiO-66 has also been proposed as a possible approach to improve the nanoscale morphology at the organic/inorganic interface [88]. The surface modification was performed using phenyl acetyl (PA), decanoyl acetyl (DA), and succinic acid (SA) in order to enhance the interaction between nanoparticles and Matrimid 5218 was used as polymer phase. A good adhesion and interaction between surface functionalized UiO-66-NH_2_ and polymer matrix was observed, leading to improved mechanical and chemical properties of and the hybrid membranes. 23 wt.% loading of PA-modified UiO-66-NH_2_ enhanced the CO_2_ permeability by 229% (from 8.5 Barrer to 28 Barrer), with a simultaneous improvement of CO_2_/N_2_ selectivity by 25%. The permeability and selectivity increased due to the strong interactions between the CO_2_ and the NH_2_ groups that are present in the MOF, together with interaction of imide group in Matrimid and aromatic ring in PA through π-π bonds. The poor interaction between fillers and Matrimid in DA and SA-modified UiO-66-NH_2_ resulted in a reduction in permeability and selectivity when compared to PA-modified UiO-66-NH_2_ particles.

The influence of amino and carboxylic group functionalization of UiO-66 have been investigated using PIM-1 as polymer phase [89]. The investigation considered “as-cast” and “solvent exchanged” PIM-1 membrane: the latter showed higher CO_2_ permeability (8210 Barrer) compared to the pristine membrane (4770 Barrer), and the difference is attributed to the excess free volume that is generated by the solvent removal. The addition of pristine UiO-66 to the matrix generated an enhancement in CO_2_ permeability (up to 59% for the “as cast” membrane and 32% for the “solvent exchanged sample, Table 2) when compared to the pristine polymeric sample. In the case of UiO-66-NH_2_ and UiO-66-(COOH)_2_, the CO_2_ permeability also showed an increase, but with a lower extent as compared to the pristine nanoparticles. In the case of the selectivity, the parameter showed a limited variation for both CO_2_/N_2_ (decrease up to 10%) and CO_2_/CH_4_ (decrease up to 20%) upon the addition of the nanoparticles, both pristine and functionalized. Performance for CO_2_/H_2_ separation were also reported. The pristine polymer showed a CO_2_-philic behavior, which was slightly enhanced in presence of the nanoparticles (particularly in the case of UiO-66-(COOH)_2_). However, the selectivity value remains too low to be attractive for industrial separations.

**Figure 6:**
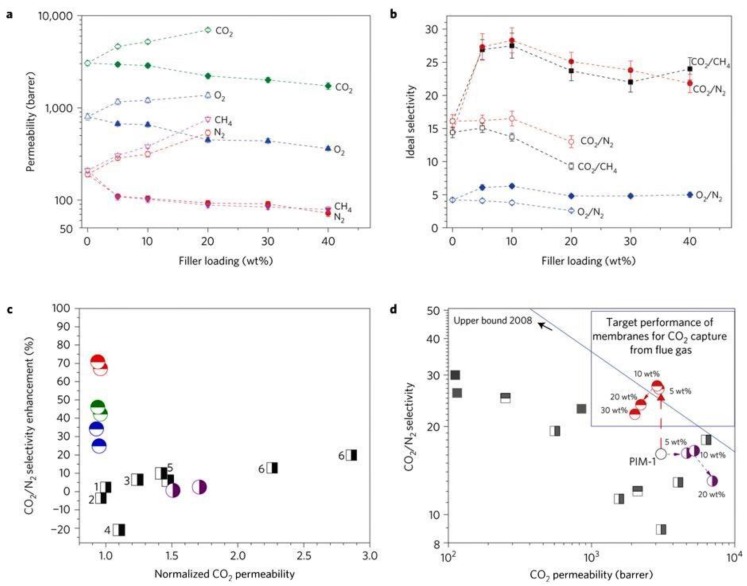
Gas permeability (**a**) and gas selectivity (**b**) of UiO-66-NH_2_ (filled symbols) and pristine UiO-66 (open symbols) embedded in PIM-1. Comparison with literature results (**c**) and Robeson plot (**d**) for CO_2_/N_2_ separation [90], with copyright permission from © 2017, Springer Nature.

In another study [90], water modulation was employed to reduce the particle size of UiO-66 (from 100–200 to around 20–30 nm) and the water modulated nanoparticles (UiO-66-H) were further surface-modified using amine (UiO-66-NH_2_) and bromide (UiO-66-Br) functional groups. The reduction in particle size improved the dispersion of UiO-66 into polymer matrix by minimizing the formation of non-selective microvoids. The decrease in the CO_2_ selective feature of the hybrids observed with increasing the content of unmodified UiO-66 was therefore prevented (Figure 6), and a selectivity enhancement was observed for all of the modified nanoparticles (up to 71% and 95% in the case of CO_2_/N_2_ and CO_2_/CH_4_ selectivity for 10 wt.% UiO-66-NH_2_ loading). This effect was mainly associated to the increased rigidity of interphase. However, the improved interactions between the functionalized nanoparticles and the polymer chains led to a negligible effect on CO_2_ permeability, which instead was significantly enhanced (~100%) in the case of unmodified UiO-66. Despite the differences that were observed with respect to the previous study in terms of pristine PIM-1 transport properties, the performance achieved by embedding UiO-66 and UiO-66-NH_2_ are similar, supporting the consistency of the results.

The influence of UiO-66 on the gas separation performance of 6FDA-based polyimides were also evaluated for mixed gas feed CO_2_/CH_4_ (50/50 *v/v*) [91]. Different 6FDA-based polymers were investigated (6FDA-BisP, 6FDA-ODA, and 6FDA-DAM). CO_2_ permeability was found to increase proportionally to the inorganic content for all of the different polymer phases, even though a larger enhancement was observed for the low permeable ones. In the case of 6FDA-Bisp and 6FDA-ODA, CO_2_ permeability improved by 357% and 178%, whereas for 6FDA-DAM, the enhancement was limited to 136%. The permeability improvement was associated to a FFV increase upon the incorporation of the inorganic phase, and the benefits was more pronounced for the polymer phase with an initially lower FFV. Improvement in terms of selectivity was observed for 6FDA-BisP and 6FDA-ODA up to 17 wt.% loading, but at higher loadings, poor nanoparticles dispersion determined a drop in the selective feature of the hybrids. Interestingly, a negligible effect was observed for the more permeable 6FDA-DAM. The authors also investigated the effect of surface functionalization of UiO-66 when embedded in 6FDA-DAM [92]. The amino-functionalized UiO-66-NH_2_ was prepared via the direct synthesis method, and UiO-66-NH-COCH_3_ was synthesized via post-synthetic modification of UiO-66-NH_2_ using acetamide-ligand. When compared to the results that were obtained with the pristine UiO-66, the surface modification helped in achieving a better polymer-MOF interface, reducing the free volume of the hybrid matrix at a given loading. At low pressure, negligible effects were observed on the transport properties when the modified MOFs were used, but at higher feed pressure, the post-synthetic modification showed better results in terms of CO_2_/CH_4_ selectivity.

In view of the reported data, UiO-66 appeared to be a promising inorganic phase to fabricate CO_2_-selective hybrid membranes. Unlike the case of ZIF, the loading for UiO-66-based mixed matrix membranes has been limited to 40 wt.%, as agglomeration and poor polymer-fillers interface was observed at high loadings. In the case of unmodified particles, the CO_2_ permeability was found to increase proportionally to the inorganic content for all of the investigated studies, but when considering the selective feature, an optimum is observed for a loading range between 10 and 20 wt.%. Amine modified UiO-66 (UiO-66-NH_2_) showed typically better performance as compared to the pristine nanoparticles, which is mainly due to the enhanced CO_2_-philicity. In general, surface modification led to improved polymer-particle interface, but for highly permeable polymers, this led to limited effect in terms of both selectivity and permeability. 

### 2.3. Copper-Based MOFs

When compared to other metal organic frameworks, Cu-based MOFs offer an exceptional CO_2_ uptake due to their high affinity with polar molecules. The presence of unsaturated open metal sites in Cu-based MOFs after activation is reported as an assisted mechanism in CO_2_ sorption [93]. Comparison of the CO_2_ adsorption capacity of two well-known MOFs containing the same ligand in their framework (Cu-BTC and Fe-BTC) showed that the Cu-BTC is characterized by a much larger CO_2_ uptake (73.2 cm^3^ g^–1^, at room temperature and atmospheric pressure) when compared to the Fe-BTC (15.9 cm^3^ g^–1^) [94]. The results clearly pointed out the higher CO_2_ affinity and interaction of Cu ions together with open metal sites, making Cu-based MOFs interesting for the fabrication of mixed matrix membranes for CO_2_ separation.

Basu et al. [49] investigated the effect of Cu_3_(BTC)_2_ when embedded in Matrimid 9725 polymer phase. Upon the incorporation of the nanofiller, both CO_2_ permeability (196%) and CO_2_/CH_4_ separation factor increased along with the inorganic content (Table 3). The overall increase of the separation performance was ascribed to the interactions between polymer and MOF and electrostatic interaction between the MOF and gas molecules, which leads to the existence of a competitive behavior. Cu_3_(BTC)_2_ was dispersed also in poly(2,6-dimethyl-1,4-phenylene oxide) (PPO), reducing the particles size via sonication from 50 to 6 µm [95]. Sonication was also reported to be able to improve the micropore volume of the nanoparticles and their dispersability within the polymeric matrix. By embedding 10 wt.% of Cu_3_(BTC)_2_ filler, the CO_2_ permeability increased proportionally to the filler content, and the maximum enhancement was achieved for the smallest particles (26%, Table 3). The reduction of particles size showed a positive impact on the membrane selectivity: the improved compatibility with the polymer matrix prevented the selectivity drop observed as in the case of bigger particles. Abedini et al. [96] embedded Cu_3_(BTC)_2_ with a particle size of 100 nm in poly(4-methyl-1-pentyne) (PMP). By increasing the loading to 20 wt.%, they observed a simultaneous increase in CO_2_ permeability (90%, Table 3) and selectivity (between 40 and 60% for the investigated gas pairs, Table 3). The observed variation was mainly attributed to a free volume increase. Interestingly, they also observed a reduction of the physical aging influence. Amine modification of Cu_3_(BTC)_2_ has also been reported as a possible approach to improve the CO_2_ separation performance of a PEBAX 1657 [97]. In view of the H-bonding between the -NH_2_ group and the polymeric chain, the modified nanoparticles showed better compatibility with the polymer phase. The CO_2_ permeability increased proportionally to the loading (up to 100% increment, Table 3) similarly for both of the fillers, but better improvement of the CO_2_/CH_4_ selectivity was achieved upon amine-modification of the fillers. Interactions between the amine groups and the CO_2_ have also been suggested to be responsible for the improved CO_2_-philicity of the hybrid matrices.

Metal-organic polyhedral 18 (MOP-18) was also used to fabricate the hybrid membrane using Matrimid as polymer phase [98]. The inorganic content was increased up to 80 wt.%, but above 44 wt.% the samples’ brittleness did not allow for the investigation of the transport properties via permeability testing. The CO_2_ permeability increased along with MOP-18 content, even though a reduction in ideal selectivity was observed for both CO_2_/N_2_ and CO_2_/CH_4_. The permeability enhancement was attributed to increasing the number of alkyl chains, which improved the CO_2_ solubility within the hybrid matrix. H_2_ permeability was also measured and the addition of the nanoparticles increased the CO_2_-philicity of the mixed matrix (Table 3). 

Ahmadi et al. [6] synthesized a new class of Cu-based microporous metal-imidazolate framework (MMIF) and explored the separation performance of the mixed matrix membranes with 10 wt.% and 20 wt.% loading in Matrimid 5218 polymer matrix. The gas permeability showed a moderate increase (26%) along with the MOF content with limited effect on CO_2_/CH_4_ and CO_2_/N_2_ selectivity. The single gas permeation results revealed a flexible structure of MMIF, with the consequent formation of interfacial defects and voids. Interestingly, a significant enhancement of the separation factor was measured for mixed gas experiments (Table 3), which was mainly attributed to CO_2_ competitive sorption within the hybrid matrix. Molecular simulation revealed that gas sorption was the dominant mechanism in the hybrid membrane, and the preferential CO_2_ uptake into the MMIF pores limited the transport of other gases (CH_4_ and N_2_) through the MMIF’s framework.

Zhang et al. [99] fabricated a Cu-based microporous metal-organic framework (Cu-BPY-HFS) and dispersed it in Matrimid 5218 up to 40 wt.% loading. The SEM images proved the good adhesion between MOF and Matrimid for loading up to 30%, but a higher amount of MOFs generated the formation of a poor particle-polymer interface. The CO_2_ permeability increased along with the Cu-BPY-HFS content, and the variation was attributed to the 0.8 Å pore diameter and the presence of interfacial voids. Interestingly, the MOF was shown to have better affinity with CH_4_ than CO_2_, and enhanced CH_4_ transport was observed in both pure and mixed gas tests. On the other hand, the CO_2_/N_2_ selectivity was negligibly affected by the presence of the MOF. As for the previous case, the addition of Cu-based MOF nanoparticles increased the CO_2_-philicity, thus reducing the ability of the hybrid membranes to separate H_2_ from CO_2_.

According to the results that were obtained from hybrid membranes containing different Cu-based MOFs, the high CO_2_ uptake capacity leads to high CO_2_ permeability. Although the increase in the gas transport is proportionally to the MOF content, a limited impact is observed on the selective feature of the hybrid membranes. Interestingly, this effect is more evident when the performance are evaluated through mixed gas permeation, which is presumably due to competitive sorption phenomena. In addition, amine modification appears to be a promising approach to improve the CO_2_-philic nature of the mixed matrix membranes.

### 2.4. Materials Institute Lavoisier MOFs (MILs)

Material Institute Lavoisier (MILs) is a sub-family of MOFs that is based on trivalent metals strongly bonded to oxygen-anion-terminated linkers. MIL-53 (chemical formula: M(OH)(O_2_C−C_6_H_4_−CO_2_, M = Al^3+^, Cr^3+^) is made of dicarboxylate ligand interconnected by octahedral chains of MO_4_(OH)_2_ and has a 3D porous structure with one-dimensional (1D) diamond-shaped channels [100]. Furthermore, it is characterized by a pore limiting diameter of ~8.5 Å and surface area (Langmuir) of 1500 m^2^/g [100,101]. Porous terephthalate MIL-53 showed a promising potential for H_2_ storage and CO_2_ capture [101,102,103,104,105,106,107]. CO_2_ adsorption experiments showed that MIL-53 exhibited a two-step sorption isotherm, behavior that was associated to gate opening phenomena. Notably, even though this type of phenomena is typically observed in MOFs at low pressure, in the case of MIL-53, this happened for pressure above 5 bar, determining the two-step shape of the sorption isotherm [100]. Depending on the metal coordinate, different breathing mechanisms have been observed: upon dehydration, for example, MIL-53(Cr) and MIL-53(Fe) have open-pore and closed-pore structures, respectively.

Dorosti et al. [108] incorporated MIL-53 in Matrimid 5218 up to 20 wt.% loading. Strong interactions of CO_2_ molecule with the oxygen atom of hydroxyl groups present in the MIL-53 structure and the breathing effect resulted in an enhancement of CO_2_ permeability proportional to the MOF loading (Table 4). The CO_2_/CH_4_ selectivity showed a significant enhancement from 31 to 51.8 between the 10 and 15 wt.% loading. However, the formation of unselective voids at higher MIL-53 content led to a significant drop of the separation performance. In view of the breathing mechanism that is affecting the MOF framework, for pressure below 3 bar MIL-53 was found to be in its close-form, whereas at higher pressure an open-framework configuration was suggested. Higher MIL-53 contents (33.3 and 37.5 wt.%) in Matrimid were investigated by Hsieh et al. [109]. They investigated the effect of the reversible structure (closed or open form) on the transport properties. In this regard, MOF dehydrated with high temperature treatment (MIL-53-ht) and characterized by an open-pore structure was compared with as synthesized nanoparticles (MIL-53-as), which presented a closed-pore configuration. At a given loading (37.5 wt.%) MIL-53-ht showed a higher permeability when compared to the as synthesized MIL, but the selective features were significantly compromised in the open-pore configuration. CO_2_/CH_4_ selectivity as high as 90.1 for CO_2_/CH_4_ was reported for MIL-53-as (Table 4). The significant enhancement was due to the sieving effect produced by the partial blockage of the pores by the BDC linkers, which prevents the permeation of molecules with larger kinetic diameter (CH_4_ and N_2_). To further prove the effect of the pore structure, MMMs containing MIL-53-lt (activated at 50 °C) were shown as a framework transition from close pore form to open pore. 

Abedini et al. [110] loaded amine-functionalized MIL-53 (Al) (100 nm size) in Poly(4-methyl-1-pentyne) (PMP) and prepared mixed matrix membranes with loading up to 30 wt.%. Addition of NH_2_-MIL-53 into polymer matrix enhanced both CO_2_ permeability and CO_2_/CH_4_ selectivity (Table 4), which is mainly due to improved CO_2_ solubility in the hybrid matrix. At higher loading, the membrane performance overcame the Robeson upper bound for CO_2_/CH_4_ separation. In mixed gas, the same trend was observed for selectivity and permeability. However, lower separation performance (10% lower permeability and 30% lower selectivity) were observed when mixed gas conditions were investigated, which is possibly due to competitive sorption phenomena. Interestingly, it was observed that the addition of the porous nanoparticles increased the CO_2_-philicity of the hybrid membranes, decreasing the H_2_/CO_2_ selectivity that was observed for the pristine polymer. MIL-53 and amine functionalized NH_2_-MIL-53 (Al) have also been dispersed in Poly(vinylidene fluoride) (PVDF) [111] and modified PVDF [112]. The modification of PVDF by means of KOH and KMNO_4_ appeared to enhance the effect of the nanoparticle to a significant extent (Table 4). The CO_2_ permeability doubled and the CO_2_/CH_4_ selectivity showed a notable enhancement (+50%) at 10 wt.% loading, with a minor effect being observed for modified and unmodified nanoparticles. In the case of pristine PVDF, a 50% enhancement of the CO_2_ permeability was associated with small influence on the selective feature of the hybrid membranes. 

Aiming at improving the interfacial interaction between MIL-53 and the polymer matrix, Tien-Binh et al. [113] introduced hydroxyl group into 6FDA-DAM polyimide backbone. 6FDA-(DAM)-(HAB) x:y copolymer (x and y denoted the copolymer ratio) containing hydroxyl groups facilitated the dispersion of MIL-53 (Al) and NH_2_-MIL-53(Al). Single gas and mixed gas (CO_2_/CH_4_ 50:50) separation performances were investigated for the mixed matrix membranes varying the copolymer ratio. Gas permeation characterization showed that the incorporation of pristine MIL-53 resulted in an increase in CO_2_ permeability for both homopolymer and copolymers with increasing the MIL loading, with the effect becoming more influential for the low permeable samples (i.e., increasing the DAM/HAB ratio, Table 4). The formation of interfacial voids is suggested to be responsible for the observed variations. On the other hand, when modified MIL-53 was used a different behavior is observed: a minimum was observed for the CO_2_ permeability at 15 wt.% loading, whereas the CO_2_/CH_4_ selectivity was optimized at 10 wt.% loading. In view of the favorable interactions between the hydroxyl and the amine group, the increase in selectivity became more significant at a higher DAM/HAB ratio, also reducing the negative effect on the CO_2_ transport across the hybrid membranes. SEM images supported this observation. Zhu et al. [114] investigated the performance of thin film hollow fiber mixed matrix membranes filled with post-modified MIL-53 (P-MIL-53). Asymmetric hollow fibers (Ultem) coated were used as support and PDMS containing different MIL-53 content was used as selective layer. The obtained results showed that the membranes containing 15% P-MIL-53 showed the best performance: the CO_2_ permeance was improved from 30 GPU to 40 GPU when compared to hollow fiber membranes coated with only pure PDMS. At 15% loading, the ideal selectivity increased from 23.3 to 28.1 for CO_2_/N_2_ and from 27 to 32 for CO_2_/CH_4_. This was mainly attributed to the strong affinity with CO_2_ due to dipole-quadrupole interaction of CO_2_ molecules with NH_2_ groups in the MOF. At 20 wt.% loading, a decrease in CO_2_/N_2_ and CO_2_/CH_4_ ideal selectivity was observed, which is mainly ascribed to particle agglomeration.

MIL-101 is another MOF from the MILs’ family, widely studied for gas separation application [115]. The MIL-101 framework is composed of chromium atoms making an octahedral framework with oxygen atoms and 1.4-benzene dicarboxilate (BDC) ligands. The rigid terephthalate ligand together with trimeric chromium octahedral clusters provides window aperture of 8.5 Å and accessible large cages. The gas sorption analysis showed that a Langmuir surface area of 5900 m^2^/g [115]. Similar to MIL-53, the removal of water molecules from the structure leaves unsaturated open metal sites in the MIL-101 structure [101]. 

Naseri et al. [116] recently reported the gas separation performance of hybrid Matrimid membranes containing MIL-101 (Cr) up to 30 wt.% loading (10 bar and 35 °C). The presence of MIL-101 in the polymer matrix enhanced the CO_2_ permeability (Table 4), with the main contribution coming from the increase in CO_2_ solubility within the hybrid matrices. The ideal CO_2_/CH_4_ and CO_2_/N_2_ selectivity showed a maximum at low loading (10 wt.%) and the drop of selective features at higher loading is mainly attributed to the presence of non-selective voids at the polymer/particles interface. The effect of addition of MIL-101(Cr) on the separation performance of a blend of Matrimid and PVDF was investigated by Rajati et al. [117]. 3 wt.% PVDF in Matrimid was selected as the most suitable blend composition for CO_2_/CH_4_ separation, which showed higher CO_2_ permeability (28%) and selectivity (22%) when compared to pristine Matrimid. The embedment of 10 wt.% MIL-101 showed a similar effect on both the pristine polymer matrix and the polymer blend, with about 60% increase in CO_2_ permeability and 40% higher selective features. The simultaneous enhancement of permeability and selectivity suggested the presence of a proper interface morphology. Additionally, the electrostatic interaction of functional groups in MIL-101 with CO_2_ resulted in better affinity and higher solubility.

MOFs-derived porous carbons (PC) based on MIL-101(Cr) and MIL-53(Al) were prepared by soaking the MOFs into NH_4_OH and carbamide, followed by calcination at 800 °C [118]. The carbonized MOFs were embedded into PPO-PEG at a loading range between 5 and 25 wt.%. For both nanoparticles, limited changes in CO_2_ permeability were observed up to 20 wt.% loading, but a marked improvement was observed at 25 wt.% loading, achieving promising permeability values (Table 4). MIL-101(Cr)-PC showed a better performance (1896 Barrer) when compared to MIL-53(Al)-PC (1266 Barrer). The selectivity showed also an improvement, and the optimum at 20 wt.% MIL content clearly suggested that higher loading probably generated interfacial voids and particle agglomeration. However, unlike the effect on permeability, MIL-53(Al)-PC showed higher selective feature when compared to MIL-101(Cr)-PC.

An interesting approach to optimize the performance of mixed matrix membranes is represented by the use of mixed MOFs [119]. A mixture of MIL-101/ZIF-8 was homogenously dispersed in PSF and no agglomeration was observed. The MMMs performance showed an enhancement in CO_2_ permeability as a function of filler loading, and the simultaneous presence of both MIL and ZIF nanoparticles showed a synergetic effect. At 35 wt.% MOF loading, the CO_2_ permeability was significantly increased (six-fold) when compared to the pristine PSF, from 5 Barrer to 30 Barrer. This was explained as increasing free volume of polymer associated to a disruption of the polymeric chains, together with the larger pore size of MIL-101. At an intermediate loading, 16 wt.%, the CO_2_/CH_4_ separation factor was increased from 23 to 40 as compared to pristine PSF. Higher loading, 35 wt.%, led to a selectivity drop, due to poor interface morphology. The authors suggested that the coexistence of ZIF-8 and MIL-101 improved the dispersion and avoided agglomeration at low particles loading. 

Finally, an interesting use of MILs as MOF scaffold (MS) has been proposed by Xie et al. [120], where the separation performance of membranes obtained from MOFs and PEG (MSP) were investigated for post combustion CO_2_ capture (CO_2_/N_2_ 10/90). Firstly, the MS membranes were fabricated on a support; then, coatings with different PEG concentration were applied to prepare the MSP membranes. The MS membranes showed extremely high CO_2_ permeance (85000 GPU), but no selective feature. Upon the application of PEG coating (PEG concentration > 0.6 mmol/5mL aqueous solution) suitable selectivity value (>30) were achieved, maintaining high CO_2_ permeability (>2700 Barrer). It was suggested that the coated polymer provides a defect free membrane and a shorter path for CO_2_ transport.

Similar to the previous MOFs, the MILs’ family also represents a group of nanoporous particles that is suitable for the development of mixed matrix membranes for CO_2_ capture. The CO_2_ permeability is frequently found to increase along with the loading, but a loading range between 10 and 15 wt.% appears to be the one that is able to optimize the selective feature of the hybrid membranes. Favorable interactions with the polymeric matrix act in the direction of enhancing the CO_2_-philicity of the mixed matrix membranes. The closed-pore structure appears to be the most suitable one for the achievement of improved separation performance; whereas, the open-pore structure is expected to enhance the gas transport through the hybrid matrix, thus possibly compromising the selectivity.

### 2.5. Other MOFs

Fe-BTC is reported to be a low cost and water stable MOF type that exhibits a pore size between 5.5 and 8.6 Å and a relatively higher surface area when compared to its Cu counterpart. Despite the lower uptake capacity when compared to Cu-BTC, the presence of a large number of coordinatively unsaturated sites and high water stability make the MOF a suitable candidate for the fabrication of mixed matrix membranes for CO_2_ separation. Fine Fe-BTC particles were dispersed in Matrimid 5218 matrix to prepare hybrid membranes, and the effect of the fillers on the gas transport properties and plasticization behaviour were investigated [121]. While limited effects were observed in single gas tests (Table 5), under mixed gas and high pressure (~40 bar) conditions, the CO_2_ permeability increased by 30% and CO_2_/CH_4_ selectivity by 62% when compared to the neat polymer. The chain rigidity of the MOF also contributed to enhance the plasticization resistance of the hybrid membrane up to 20 bar. The effect of Fe-BTC filler on the transport properties of Matrimid has also been studied by Rita et al. [122]. The study revealed that gas diffusivity changes with increasing temperature dominated the drop in solubility, leading to an overall increase in CO_2_ permeability from 94.2 Barrer at 303 K to 217.9 Barrer at 353 K with a 30 wt.% MOF loading. Interestingly, the CO_2_/N_2_ selectivity of the matrix increased on a similar scale with temperature increase. The effect of Fe-BTC on rubbery PEBAX 1657 for gas permeation was studied by Dorosti and Alizadehdakhel [123]. Both single gas and mixed gas (CO_2_/CH_4_) tests revealed a four-time increase in CO_2_ permeability when compared to the neat polymer (Table 5). The gas selective feature of the hybrids showed a minor increase as compared to the pristine polymer, but a significant drop is observed at 40 wt.% loading due to the formation of non-selective voids. Differently from what has been observed for the glassy polyimide, the increase in feed pressure led to plasticization phenomena, and consequently to a drop in selectivity. 

**Figure 7:**
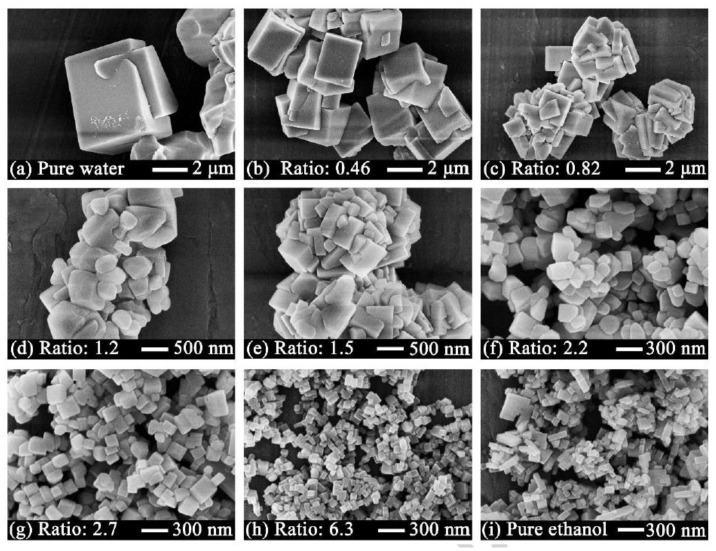
Fine-tuning crystal size of KAUST-7 by varying ethanol-water (solvent) ratios in synthesis solution: (**a**) pure water, ethanol/water ratio of (**b**) 0.46, (**c**) 0.82, (**d**) 1.2, (**e**) 1.5, (**f**) 2.2, (**g**) 2.7, (**h**) 6.3, and (**i**) pure ethanol [125], with copyright permission from © 2018 Elsevier.

A new sorption selective, chemically stable, fluorinated MOF NbOFFIVE-1-Ni (KAUST-7) was developed by Cadiau et al. [124]. KAUST-7 showed an apparent pore size of 4.75 Å and a CO_2_ sorption capacity of 2.2 mmol/g at 25 °C and 1 bar. Recently, Chen et al. [125] synthesized nanosized KAUST-7 crystals by novel co-solvent synthesis method (Figure 7) and dispersed them in 6FDA-Durene matrix. The CO_2_ permeability increased along with the loading from 750 (pristine polymer) to 1038 (33 wt.% loading) Barrer (Table 5). The selectivity marginally increased due to both increase in solubility selectivity and diffusivity selectivity. Additionally, interactions between the organic ligand and the groups of 6FDA increased compatibility, leading to enhanced plasticization resistance up to 10 bar, with a minor reduction in CO_2_/CH_4_ selectivity of 33% MOF loaded matrix. 

Bimetallic MOFs, like Mg_2_(dobdc), contain many open metal sites along the pore walls facilitating a selective adsorption and transport of CO_2_. Bae and Long [126] developed a facile synthesis method to produce 100 nm primary crystals of Mg_2_(dodbc) and successfully incorporated them in three different polymer matrices: PDMS, crosslinked-PEO, and 6FDA-TMPDA (polyimide). The study revealed that the MOF had a negative effect on the gas transport through the rubbery polymers (Table 5), possibly due to the plugging of the MOF pores by the rubbery polymer chains. On the other hand, a simultaneous enhancement of both CO_2_ permeability and CO_2_/N_2_ selectivity was observed for the glassy polyimide (Table 5). It was shown that the variation was mainly associated to the increase in CO_2_ solubility, with minor effects on the gas diffusion through the selective layer. A similar study by Smith et al. [127] proved that the addition of Mg_2_(dobdc) to 6FDA-Durene increased the permeability of CO_2_, N_2_, H_2_, and CH_4_ due to the increase in diffusivity of the penetrants. It was observed that the MOF particles further increased the brittleness of the films due to densification. By changing the coordination site, Ni_2_(dodbc) was fabricated and it was found to improve the mechanical robustness, owing to smaller primary particle size. Both bimetallic MOFs were found to improve the performance in separations governed by diffusivity differentials, like H_2_/CH_4_ and H_2_/N_2_ when compared to CO_2_/CH_4_ and CO_2_/N_2_ separations that require both solubility and diffusivity enhancement.

## 3. Porous Organic Frameworks (POFs)

**Figure 8:**
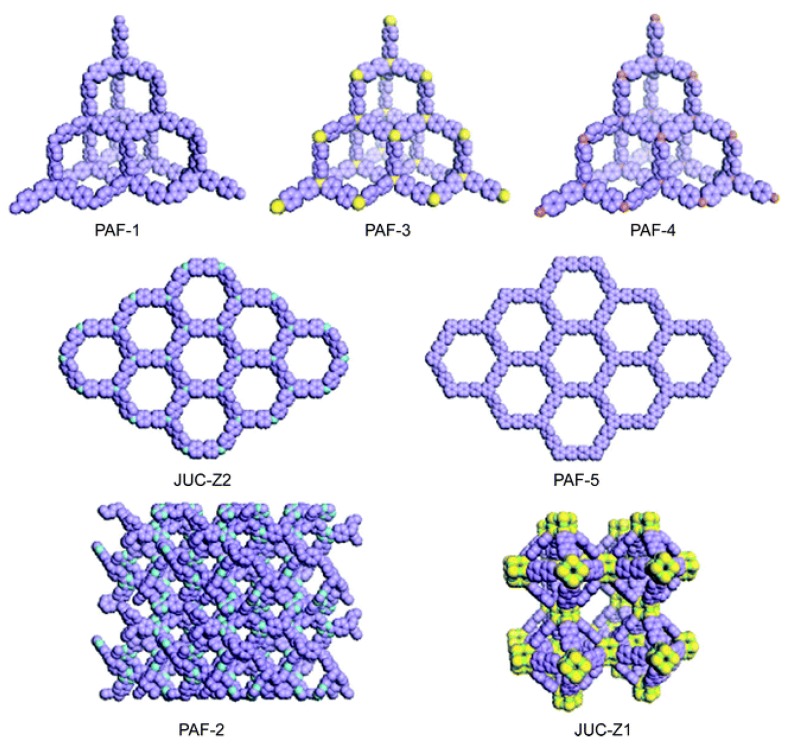
Structure model of synthesized and simulated porous aromatic frameworks. Atom colors: C = purple, N = blue, Si = yellow, O = green, Ge = brown [134], with copyright permission from © 2012, Royal Society of Chemistry.

Metal-organic frameworks have drawn considerable attention for their tunable chemistry and gas separation and storage performance, many MOFs suffer from the lack of chemical and physical stability. In addition, their limited sorption capacity and the presence of heavy metal ions in their framework have posed obstacles for their prospective applications [128]. Recently, a new class of porous materials known as porous organic frameworks (POFs) has attracted great attention as an alternative to MOFs. POFs can be either crystalline, such as covalent organic frameworks (COFs), or amorphous with uniform pore diameter, such as porous aromatic frameworks (PAFs) [129,130]. Due to the entirely organic structure, POFs ensure good adhesion with organic polymer phase and display better chemical compatibility [129]. PAF-1 (Figure 8) was synthesized and characterized for the first time in 2009, with the scope of exploring its potential as adsorbent [131]. PAFs have a local diamond-shape with tetrahedral bonding of tetraphenylene methane in their main building block. The exceptional surface area (Langmuir surface area of 7100 m^2^/g) of PAFs has shown excellent sorption capacity for hydrogen and carbon dioxide (i.e. 1300 mg/g CO_2_ uptake at 25 °C and 40 bar). Furthermore, they are characterized by super hydrophobicity, enhanced adsorption enthalpies, and delocalized charged surface [128,131]. Thermal analysis of PAFs exhibited that the structural integrity remained intact up to 520 °C in air and after water boiling point for seven days [131]. The pore size distribution of PAF-1 displays a pore diameter of 1.4 nm, which can be tuned via activated carbonization to 0.79, 0.93, 0.64, and 0.6 nm while using KOH, NaOH, CO_2_, and N_2_ as an activation agent, respectively [132]. Furthermore, a Monte Carlo simulation study suggested that a nitrogen-doped PAF (NPAF-11) containing imidazolic group improves the CO_2_ uptake more than 130% when compared to PAF-1 [133].

The non-equilibrium nature of glassy polymers makes them subject to physical aging, which tends to reduce their fractional free volume over time, and thus, the gas permeability coefficient. Porous organic frameworks have been reported to have the ability to play as an anti-aging filler, as they can freeze the nanostructural morphology, slowing down the aging process to a significant extent. Lau et al. [135] embedded PAF-1 (10 wt.% loading) into three high free volume glassy polymers including PTMSP, PIM-1, and PMP in order to explore the influence of porous organic fillers on aging process of these polymers. Over a period of eight months (240 days), the CO_2_ permeability of pristine PTMSP dropped from 20,000 Barrer to a value of 12,500 Barrer (37% drop). The hybrid membrane containing 10 wt.% PAF-1 showed a higher CO_2_ permeability (approximately 25,000 Barrer), which dropped of only 7% over the investigated period. Similar effects were observed for PIM-1 and PMP (Table 6). Interestingly, the CO_2_/N_2_ selectivity improved with aging, similarly to the pristine matrix. Following a similar goal, Volkov et al. [136] embedded PAF-11 in PTMSP membrane (1–10 wt.% loading) and monitored the variation of the transport properties over time through single gas permeation experiments. Initially, the addition of PAF-11 nanoparticles corresponded to an increase of the gas permeability of PTMSP, with a negligible effect on the selective features of the membranes. Long-term exposure to high temperature showed that the presence of the PAF nanoparticles helped in improving the mechanical stability of the PTMSP matrix: pristine PTMSP could not withstand more than 200 h exposure at high temperature, whereas the hybrid matrixes were tested up to 510 h, showing good mechanical properties. Furthermore, the membrane with 10% PAF-11 loading showed a limited drop of the CO_2_ permeability (30%), with stable performance over a period of more than 300 h. 

Functionalization of PAF-1 has been reported as an effective method to improve the CO_2_ permeability in hybrid membranes [137]. The presence of functional groups (NH_2_, SO_3_H, C_60_ nanoparticles, and Li_6_C_60_ composites) added to PAF-1 affected the CO_2_ sorption capacity, mainly due to the affinity of polar functional groups with CO_2_. Particularly promising is the introduction of Li_6_C_60_ functionality, which is able to provide additional CO_2_ sorption sites that are associated to the lithium, also increasing the PAF-1 surface area (from 3760 to 7360 m^2^ g^-1^). The CO_2_ permeability of PTMSP increased from 30,000 to 55,000 Barrer, and the effect of aging was limited to a 10% decrease over a period of 365 days for a 10 wt.% of PAF-1-Li_6_C_60_ loaded in PTMSP. CO_2_/N_2_, and CO_2_/CH_4_ selectivity were affected by the addition of the nanoparticles and by the physical aging to a limited extent (Table 6). Mitra et al. [138] studied the influence of a hypercrosslinked (HPC) nanofillers on the performance of PIM-1. PIM-1 membrane, prepared using dichloromethane as solvent, showed a CO_2_ permeability of 2258 Barrer, which dropped to a value of 1109 Barrer after 150 days. A similar trend was also observed when chloroform was used as solvent (Table 6). The addition of HCP into PIM-1 reduced the effect of physical aging for the samples prepared with different solvents, but at high loadings, the selectivity was negatively affected by the presence of the nanoparticles. Interestingly, the addition of HCP was found to prevent membrane swelling in the presence of ethanol.

Despite the limited amount of investigations, POFs appeared to be promising materials for the fabrication of CO_2_ separation applications. The main advantage they add to polymeric materials is the significantly reduced physical aging phenomena; therefore, they are of interest for high free volume polymers. However, even though they are characterized by high CO_2_ uptake, their addition can increase the CO_2_ permeability (even in high free volume polymers), but has a limited or negligible effect on the selective feature of the hybrids. Interestingly, the young modulus has been reported to benefit from the addition of PAFs [136].

## 4. Zeolites

Zeolite molecular sieves are a class of aluminosilicate crystals that have been studied extensively and are one of the most widely reported porous materials for CO_2_ capture because of their physiochemical properties [139,140]. The pore size of zeolites varies from 4 Å to 1.2 nm and their frameworks are formed by interconnecting channels. The molecular sieving nature coupled with the strong dipole-quadrupole interaction with carbon dioxide make zeolites promising candidates for carbon capture. Si and Al derived from silicate compounds are the main building block of zeolites nanoparticles. The morphology is controllable by varying the Si and Al content, as changes in the Si/Al ratio lead to the electrostatic charge variation, resulting in different pore sizes distribution and adsorption capacities [139]. The thermal and chemical stability of zeolites can be improved by increasing the Si content [139], even though the zeolites do not provide the level of tenability offered by MOFs [129,140]. Zeolites of interest for CO_2_ capture applications are classified into three main categories: zeolites with small pore size (Linde Type A, LTA), medium pore size (Mordenite Framework Inverted, MFI), such as ZSM-5, and large pore size (Faujasite, FAU). Extensive studies have been dedicated to ensure the good adhesion between zeolites and polymer phases, as interfacial defects and voids between the organic and inorganic phases frequently resulted in the poor separation performance of the hybrids [141]. Unlike MOFs, zeolites structure is rigid and the pore dimensions are generally fixed. However, their activation by calcination may have detrimental impact on their framework integrity. The absence of accessible open metal sites (hidden by oxygen ions in the zeolite structure) is responsible for a lower CO_2_ uptake when compared to MOFs [141]. The mechanism typically used to describe the transport of light penetrants through zeolites is solution-diffusion [142]. Extensive studies have been dedicated to the incorporation of zeolites in hybrid membranes for CO_2_ separation [142]. Nevertheless, the research is still extremely active, and many studies on hybrid membranes for CO_2_ applications employing zeolites have been reported in recent years. 

Hoseinzadeh Beiragh et al. [143] investigated the effect of ZSM-5 loading on the CO_2_/CH_4_ separation performance of PEBAX-based membranes. The single gas permeation results revealed that an optimum for CO_2_ permeability is achieved at low zeolite content (5 wt.%, Table 7), whereas the CO_2_/CH_4_ selectivity increased proportionally to the zeolite loading, achieving a 67% enhancement when compared to the pristine polymer. The sieving effect of zeolite (pore diameter 5.4 Å) has been suggested to be the main reason for the enhancement of the separation performance, and the decrease in fractional free volume was identified as explanation of the permeability drop at high zeolite contents. Interestingly, at higher feed pressure (up to 5 bar) the beneficial influence of zeolites on the mixed matrix performance is significantly reduced. Contrasting results were obtained when ZSM-5 have been embedded in a glassy polyimide (Matrimid 5218) [144]. In this case, the permeance increased along with the particle loadings (from 5 to 21 GPU), whereas the selectivity showed a 75% decrease. The results have been mainly associated to poor compatibility between ZSM-5 and Matrimid, which resulted in particles agglomeration and the presence of interfacial voids already at low loadings. 

Zeolite 13X have been used by Bryan et al. [145] to prepare hybrid membranes based on PEBAX 1657. When compared to ZSM-5, X zeolites are characterized by a larger pore size, between 11 and 14 Å [146]. The addition of 15 wt.% nanoparticles in the polymer matrix led to the improvement of both CO_2_ permeability (from 81 to 114 Barrer) and CO_2_/N_2_ selectivity (from 41 to 47), suggesting the achievement of proper interface morphology between the particles and the polymer matrix. Surya Murali et al. [147] have also prepared mixed matrix membrane using PEBAX 1675 as polymer phase. Zeolite 4A was embedded up to 30 wt.% in the polymeric matrix, showing agglomeration at higher loading. A 3-fold enhancement of the CO_2_ permeability was observed with increase in inorganic loading, but the selective feature showed an optimum between 5 and 10 wt.%, which is possibly due to the interfacial voids formation. Zhao et al. [148] fabricated mixed matrix embedding up to 50 wt.% SAPO-34 zeolite (pore diameter 3.8 Å) in PEBAX 1657. The CO_2_ permeability increased proportionally to the inorganic content, achieving three-fold enhancement when compared to the pristine polymer. The consistency with the Maxwell model prediction also suggested the achievement of a proper polymer-filler interface. However, the CO_2_-selective feature of the hybrids were negligibly affected (Table 7). Interestingly, even though SAPO-34 inorganic membranes own impressive CO_2_/H_2_ selectivity [149], the performance of the pristine PEBAX 1675 were negligibly affected for the entire loading range investigated. Rezakazemi et al. [150] investigated the influence of 4A zeolite on the transport properties of polydimethylsiloxane (PDMS). The hybrid membranes showed a proper polymer-fillers interface up to 50 wt.% loading. Interestingly, a significant H_2_-sieving effect was observed for the fabricated membranes: H_2_ permeability increased along with the inorganic content, whereas both CO_2_ and CH_4_ transport was hindered. The pristine PDMS was found to be CO_2_-selective for H_2_ separation, but at 20 wt.% 4A loading, the hybrid material showed H_2_—selective feature, suggesting that the membrane shifted from being solubility-driven to a condition where the diffusion coefficient dominates the gas transport.

Recently, Atalay-Oral et al. [151] proposed a comparative study about the effect of different zeolites on the transport properties of polyvinylacetate (PVAc). They compared four different zeolites: 4A (pore size 4.2 Å), Ferrierite (pore size 4.2 Å), 5A (pore size 5.2 Å), and Silicalite-1 (pore size 5.5 Å). For all of the different fillers, the CO_2_-selective features of the mixed matrix membranes were increased. Ferrierite showed the better improvement in terms of performance: the selective feature (both CO_2_/CH_4_ and CO_2_/N_2_) increased proportionally to the inorganic content (Table 7), whereas the permeability increased at 20 wt.% loading, but negligible differences were observed at higher inorganic content. The authors suggest the strong interactions between Ferrierite cations and CO_2_ molecules to be the main reason the superior performance of the Ferrierite-based hybrid membranes. Another study compared the performance of Zeolite A (Si/Al = 1) and zeolite ITQ-29 (Si/Al = ∞) when embedded in PTMSP [152]. Surprisingly, when single gas tests were performed on a hybrid membrane containing 20 wt.% Zeolite A loading, a 35% drop in CO_2_ permeability was observed being combined with a 70-fold enhancement of the CO_2_/N_2_ selectivity (Table 7), surpassing the Robeson’s upper bound. The extraordinary performance was attributed to the molecular sieving ability of the nanoparticles and to the achievement of a proper interface morphology. A much lower improvement was observed in the case of ITQ-29 zeolite, which is mainly due to the poor polymer-zeolite interactions and consequently interfacial voids formation. These results highlighted that choosing the proper Si/Al ratio is extremely important in the design of hybrid membranes, as it directly affects the organic/inorganic interfacial morphology. Nevertheless, the same authors reported that under mixed gas conditions, the separation factor of the Zeolite A/PTMSP membranes appeared to be lower (5) when compared to the ideal selectivity (63) [153]. The authors concluded that the influence of the diffusivity selectivity dominates the transport, rather than the preferential sorption capacity in the mixed matrix.

**Figure 9:**
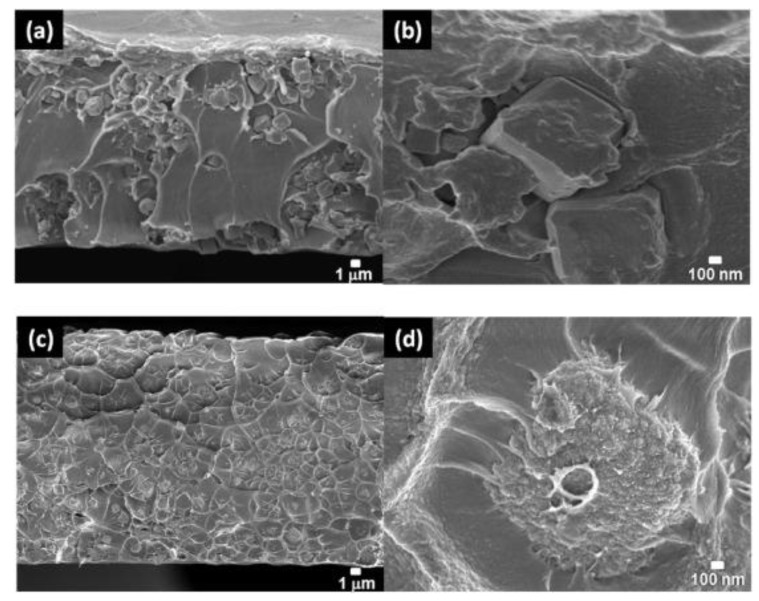
Cross section FESEM images of mixed-matrix membranes: (**a**,**b**) Matrimid with embedded pristine zeolite 5A; and, (**c**,**d**) Matrimid with embedded surface modified zeolite 5A [155], with copyright permission from © 2016 Elsevier.

As previously reported for MOFs and POFs, surface functionalization of zeolites is reported as a successful approach to improve the polymer-particles compatibility, and, thus, the membrane performance. The presence of unselective interfacial voids at the interface between zeolite 4A and PSF determined a significant drop of the CO_2_/CH_4_ separation efficiency (Table 7), without enhancing the CO_2_ permeability to a significant extent [154]. However, the functionalization of the zeolites particles with MgCl_2_ and NH_4_OH resulted in increased selectivity up to 30 wt.% loading, with a limited effect on the CO_2_ permeability. Similarly, zeolite 5A have also been modified with Mg-based moieties to improve the adhesion with the polymer chain in Matrimid-based membranes [155]. Surface treatment of zeolite with Mg(OH)_2_ improved both CO_2_ permeability (10.2 to 22.4 Barrer) and CO_2_/CH_4_ selectivity (33.6 to 36.4). As shown in Figure 9, the modification of the nanoparticles allowed for significantly improving the interface morphology between the nanoparticles and the polymer phase, preventing the formation of interfacial voids. Effect of surface modification was investigated also for zeolite NaY. Mixed matrix membrane were fabricated embedding the pristine and modified nanoparticles (loading range: 0–25 wt.%) in cellulose acetate [156]. Surface modification of zeolite with NH functional groups was performed in order to improve the CO_2_ separation performances. However, in this case, the functionalization showed minor improvement when compared to the pristine nanoparticles (Table 7). 

According to the data reviewed, the fabrication of hybrid membranes containing zeolites can be promising for CO_2_ capture applications. Loading up to 50 wt.% have been investigated, and rubbery materials (e.g., PEBAX or PDMS) showed good compatibility with the pristine nanoparticles, independently from their nature. Similar to MOFs, increasing the loading of pristine zeolites within polymeric phases enhances the CO_2_ permeability of the hybrid membranes. However, this effect is mainly observed for low permeable polymers, since when high free volume polymers are used the hybrid membranes showed lower permeability when compared to the polymeric precursor. The effect on the selective features depends on the organic-inorganic interface, but a sieving effect for CO_2_ is rarely observed. As observed for MOFs, surface modification is a suitable approach to improve the polymer-particles interface, but typically, the better compatibility mainly improves the selective features, and CO_2_ permeability appears to be negligibly affected by the presence of the inorganic phase.

## 5. Porous Nanosheets

**Figure 10:**
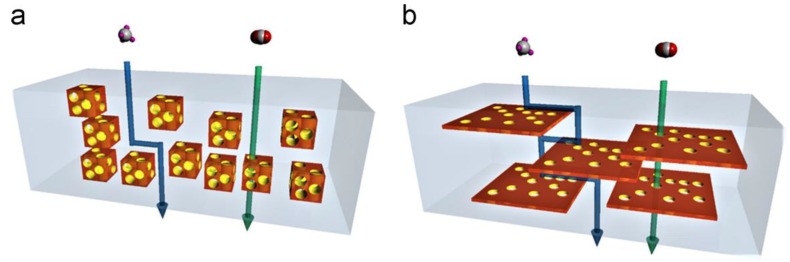
Schematic representation of the effect of isotropic particles (**a**) and nanoporous sheets (**b**) on the transport through mixed matrix membranes. Reprinted from [163], with copyright permission from © 2013 Elsevier.

Two-dimensional nanoporous nanomaterials have been of great interest owing to their layered structure, which can significantly improve the sieving effect of nanoporous materials to gas transport. Inorganic membranes that are made of 2D metal organic frameworks have been reported in literature, showing promising separation performance [157,158]. 2D structures have also been reported for zeolites [159], and inorganic membranes have been fabricated [160,161], even though their potential for gas separation remains unexplored. The high aspect ratio of two-dimensional nanoporous particles makes them extremely attractive for the fabrication of mixed matrix membranes. Layered fillers perpendicular to the concentration gradient of the gas species in the membranes can give rise to outstanding separation performance because of a significant increase in tortuosity, hence in diffusive pathways, of the penetrants that cannot permeate through the nanoporous structure (Figure 10). A comparison in the water/ethanol separation performance of ZIF-8 and its 2D derivate (ZIF-L) showed a simultaneous improvement of both permeability and selectivity at even lower MOF loading [162]. Next generation of hybrid membranes containing porous nanosheets that are incorporated in polymer matrix will provide a solution in order to enhance the separation performance of membranes for CO_2_ separation. 

Porous layered and delaminated materials, with an intermediate structure between clay-like morphology and porous frameworks, represent an interesting class of porous 2D nanofillers. These materials can be exfoliated from bulk crystals, giving rise to high aspect ratio structures containing a porous architecture that can be of interest for gas separation applications. Layered aluminophospates (AlPO), layered silicates (AMH-3), layered titanosilicates JDF-L1, and layered COFs (NUS-2/3) are some examples that have been used for the fabrication of mixed matrix membranes. Nevertheless, very few studies have been dedicated to CO_2_ separation, since selective sieving of H_2_ from bigger molecules like CH_4_ have been investigated to a bigger extent. 

A pioneering work was developed by Kim et al. [163], where nanoporous layered silicate AMH-3 (pore size 3.4 Å) was first exfoliated and subsequently embedded in cellulose acetate, achieving a loading up to 6 wt.%. The CO_2_ permeability increased along with the inorganic loading, and this enhancement was attributed to the competing effects of transport through the nanopores, the interlayer spaces, and through a lower-density cellulose acetate phase. Negligible influence was observed on the selective features. Kang et al. [164] reported novel synthesis of NUS-2 and NUS-3 layered materials that are based on COFs with excellent water and acid stability. Both of the COFs exhibit hexagonal channels with diameters of 0.8 nm and 1.8 nm for NUS-2 and NUS-3, respectively. The flower-like nanofillers contain leafs of 1–2 µm length and 50–100 nm width. The synthesized nanofillers were dispersed in two different polymer matrices Ultem (PEI) and polybenzimidazole (PBI) and separation performances for H_2_/CO_2_ and CO_2_/CH_4_ were studied. For CO_2_/CH_4_ separation with Ultem, both of the nanofillers increased CO_2_ permeability and selectivity at 10 and 20 wt.% loading moving the pristine polymers closer to the upper bound. However, when the filler content was increased to 30 wt.%, the selective features of the membrane dropped, possibly due to void formation at the polymer/filler interface. On the other hand, for H_2_ separation from CO_2_, the PBI sample containing 20 wt.% NUS-2 surpassed the upper bound thanks to an impressive enhancement of the H_2_/CO_2_ selectivity. Alternatively, NUS-3 increased the permeability while maintaining or decreasing the selectivity. The highest H_2_ permeability was obtained at 30 wt.% loading, which is 17 times the permeability of pristine PBI, which is followed by a 50% reduction in selectivity. 

Rodenas et al. [165], in 2014, synthesized and compared CuBDC MOFs with three different morphologies: isotropic nanocrystals (nc-CuBDC), bulk-type crystals (b-CuBDC), and nanosheets (ns-CuBDC). The different nanoparticles were embedded within a polyimide-based (Matrimid 5218) polymeric matrix. It was shown that the CuBDC offered large surface area, which was about five-fold higher than the one that was obtained for the b-CuBDC. Mixed gas permeation tests showed that the addition of both nc-CuBDC and b-CuBDC (8 wt.% loading) determined a drop in the CO_2_/CH_4_ selectivity when compared to the pristine polymer. However, when a similar loading of ns-CuBDC was embedded in the polymeric matrix, a 30% enhancement in the separation factor was observed. This effect was even more evident when the feed pressure was increased from 3 to 7.5 bar, where the selectivity improvement reached a 80% higher value as compared to the pristine polymer. At 3 bar feed pressure, the CO_2_ permeability gradually increased from 5.78 to 9.91 Barrer (at 3.7 wt.% loading) and then decreased to 4.09 Barrer (at 8.3 wt.%, Table 8). At a similar loading of bulk and nanocrystals, a minor reduction in CO_2_ permeability was observed. Interestingly, the embedment of ns-CuBDC was also reported to limit the effect of CO_2_-induced plasticization characteristic of polyimides at high partial pressure of CO_2_. The authors attributed this effect to the depletion of MOF-free permeation pathways, sustaining the selective features of the membrane under high CO_2_ concentration within the hybrid matrix. A similar work has also been recently reported by Shete et al. [166], who embedded Cu-based MOF nanosheets (lateral size 2.5 µm, thickness 25 nm) in Matrimid 5218. Results that were obtained by the two studies are quite similar, with a decrease in CO_2_ permeability at increasing the nanosheets loading with improved selectivity (Table 8), strengthening the consistency of the influence of nanosheets on the transport properties of polyimides. The main difference is related to the influence of the feed pressure: in the latter case, the mixed matrix membranes selectivity decreased with increasing the operating pressure, whereas an opposite trend was observed in the other study.

The CO_2_/CH_4_ gas separation performance of ultrathin layer that was obtained by dispersing 2D MOFs in PIM-1 was investigated by Cheng et al. [167]. CuBDC nanosheets with a thickness of 15 to 40 nm and ~100 aspect ratio were successfully embedded into PIM-1 up to 5 wt.% loading. Thin films (200 to 2200 nm) were then coated on a porous Al_2_O_3_ support via spin-coating technique. At 10% loading, the enhancement in CO_2_ selectivity from 4.4 to 16 (~300% increase) was observed. Nevertheless, the selectivity improvement with an increase in MOF loading was at significant expense of the CO_2_ permeance, which dropped from 1750 to 500 GPU with a 2 wt.% loading. Interestingly, no significant differences in permeance have been observed between 2, 5, 10, and 15 wt.% loading, suggesting that the transport is dominated by the embedded phase already in the low loadings (Table 8). The gas selectivity enhancement was attributed to the tortuosity and the pathway created by centrifugal force, which helped to align nanosheets horizontally. At higher loading up to 15 wt.%, the selectivity reduction was observed mainly due to the presence of nonselective voids and agglomeration. 

Yang et al. [168] recently reported the influence of CuBDC nanosheets on the performance of high free volume polymers, such as PIM-1 and 6FDA-DAM. Nanosheets with a lateral dimension of 1–8 µm and a thickness of 40 nm were synthesized and embedded in the polymer phase via sonication. As observed previously for PIM-1, the incorporation of nanosheets resulted in a decrease of CO_2_ permeability at the low loadings for both PIM-1 and 6FDA-DAM (Table 8). Notably, in the case of PIM-1 small differences were observed between the two different filler loadings, whereas in the case of 6FDA-DAM, the permeability decrease was more evident between the 2 and 4 wt.% loading. In both cases, the presence of the porous nanosheets led to a significant increase (20–40%) in the selective feature of the hybrid membranes.

In another study, Kang et al. [169] prepared MMMs with a newly synthesized 2D MOF (10 × 100 nm^2^), [Cu_2_(ndc)_2_(dabco)]n, (ndc = 1,4-naphthalene dicarboxylate, dabco = 1,4-diazabicyclo[2.2.2]octane), and incorporated into PBI (polybenzimidazole) matrix for pre-combustion CO_2_ separation. MOF loading from 10 to 20 wt.% provided highly selective MMMs, with about 100% increment in H_2_/CO_2_ ideal selectivity. The authors attributed the high selective features to fast H_2_ permeation through the MOF, whereas CO_2_ follows slower diffusive pathways in view of the larger kinetic diameter. Higher MOF loadings (> 20 wt.%) resulted in a selectivity drop in selectivity, which is possibly due to void formation. Comparison of different morphologies showed that MOF nanosheets offered better selectivity and permeability of the hybrid membranes because of the shape, orientation, and interfacial adhesion inside the matrix. As in the previous case, similar loadings of bulk or nanocrystals (20 wt.%) showed lower selectivity values compared to the nanosheet morphology.

Despite the early stage of the research, the analysis of the performance achieved while using 2D nanoporous materials for the fabrication of mixed matrix membranes clearly showed a promising potential within CO_2_ capture. Systematically, the 2D shape was demonstrated to be able to achieve better performance when compared to the isotropic particles, independently from their size. Interestingly, compared to isotropic nanoparticles, the effect of nanosheets is already visible in the low loading range, similar to what has been observed for graphene [22]. The use of 2D porous nanoparticles can be of particular interest for the enhancement of the selective feature of high free volume polymers, where a partial loss in CO_2_ permeability can be tolerated if being counterbalanced by a significant enhancement of the separation factor. A notable increase of studies that are dedicated to this topic is expected in the near future.

## 6. Conclusions and Perspective

The recent advances in the synthesis and improvements of 2D and 3D porous nanophases has driven a continuous research within the development of mixed matrix membranes for gas separation purposes. In particular, the possibility of tuning the pore diameter to a gas-sieving level and the CO_2_-philicity of the pore cavity has the potential to facilitate the simultaneous enhancement of the solubility and diffusivity coefficient of carbon dioxide. Therefore, CO_2_ permeability and selectivity can be expected to benefit from these features, leading to a shift in the separation performance towards the upper right corner of the Robeson plot. 

Notable attention has been given to MOF nanoparticles and MOFs nanosheets. The pore opening size falling within the gas kinetic diameters and the presence of unsaturated open metal sites makes them of particular interest for CO_2_ separation. Analysis of adding ZIF nanoparticles into highly or moderately permeable polymeric membrane materials reveals a clear tendency to improve the CO_2_ permeability when the nanofiller loading is increased to 30–40 wt.%. The incorporation of ZIFs has been frequently reported to be associated to the disruption the polymer chain packing, leading to an increase of the MMMs free volume. However, selectivity enhancement was seldom reported despite the expected sieving effect and the observed suitable interface morphology. The ZIFs flexible framework is expected to be among the main reasons for this phenomenon. Also, in the case of other analyzed MOFs (UiO-66, MILs and various metallic-based MOFs), the CO_2_ permeability enhancement was frequently observed, with the enhancement being proportional to the MOF content. However, the increase in selective feature was typically reported only at low particles loading (especially for UiO-66) and mild operative conditions, suggesting that the sieving ability of the pore opening is not extremely effective for gas separation purpose. Structural flexibility and poor interface interactions were frequently mentioned as the possible causes. Therefore, the achievement of a more rigid structure of the MOFs cage and more effect functionalization are desirable to improve the efficiency of the embedded phase. Interestingly, particles with smaller size have shown to be more effective compared to inorganic phases with bigger size. In addition, particles with reduced size can facilitate the fabrication of thin (<1 µm) selective layers.

Porous nanosheets showed a promising potential for the fabrication of mixed matrix membranes for CO_2_ separation. When compared to 3D porous materials, the impact of 2D nonoporous materials is demonstrated even at low loading range (<10 wt.%). The use of 2D shape was systematically demonstrated to obtain better performance compared to isotropic particles. Higher selectivity can be achieved using MOF nanosheets, even when they are incorporated in high free volume polymers, but the variation typically takes place to the expense of the gas transport through the selective layer. The intrinsic nature of these 2D nanoparticles has the potential to be a successful strategy to efficiently fabricate mixed matrix membranes with superior separation performance in the form of thin composite membranes. Therefore, future work has to focus on the reduction of the thickness of these 2D porous layers, allowing for achieving membrane thickness in the order of 100–200 nm.

Porous organic frameworks (POFs) have also been recently investigated for CO_2_ separation. Their fully-organic nature facilitates their dispersion in polymer phases, but their more rigid structure confers interesting feature to the hybrid membranes. Experimental results gave evidence of an unprecedented capacity of stopping physical aging in high free volume polymers. Even though CO_2_ permeability is frequently enhanced using PAFs, negligible influence on selectivity of the hybrids was observed. Nevertheless, their promising performance has been disclosed only for thick self-standing membranes, and more investigation on their efficiency for thin films are needed to fully understand their potential.

Zeolites, as one of the most common fillers, attracted great interest in MMMs fabrication and have been investigated for last two decades. When compared to MOFs, the absence of organic ligand in the lattice, the control of zeolite/polymer interface is more difficult than MOF/polymer interface. Therefore, many efforts have been spent to ensure the achievement of proper interface morphology to reduce the negative effects that are associated to interfacial voids. Loading of up to 50 wt.% has been reported, and rubbery polymers (e.g., PDMS) showed good compatibility and adhesion with pristine nanoparticles. Increase in zeolite content led to higher permeability and effect of pristine zeolites on CO_2_ permeability was more pronounced for low permeable polymers when compared to high free volume polymers. Surface modification of zeolites have shown better compatibility and improved selectivity with negligible effect in CO_2_ permeability.

The following focuses may be of appreciable impact in the future development of MMMs with superior transport properties:

- to reduce primary particle size of existing MOFs and expedite their incorporation in thin composite polymeric membranes;
- to increase the CO_2_ affinity and the polymer/particle interactions by novel surface functionalization procedures on the nanoparticle or by post-functionalization after membrane preparation, aiming at improving the CO_2_ separation performance and simplifying their dispersion in the polymeric phases;
- to tune the structure and morphology of POFs with the aim of enhancing the selectivity of hybrid matrix when used in high free volume polymers;
- to design and fabricate novel 2D MOF frameworks with improved sieving ability that do not sacrifice the gas transport through the selective layer; and,
- to systematically investigate the potential of hybrid membranes in H_2_ separation, exploiting the exceptional H_2_ sieving ability of some MOFs.

## Figures and Tables

**Table 1:** Gas separation performance of ZIFs-b ased mixed matrix membranes (operating conditions ranging within 1–5 bar, 20–35 °C, unless differently specified).

Filler	Polymer	Loading (wt.%)	P_CO2_ (Barrer)	α _C__O2__/__N2_	α _C__O2__/__CH4_	α _CO__2__/__H2_	Ref.
ZIF-8							
	Matrimid 5218	0	9.5	30.7	39.8	0.34	[48]
ZIF-8		20	9.0	30.1	51.1	0.29	
50–150 nm		30	14.2	24.1	38.2	0.31	
		40	24.5	23.4	27.8	0.35	
		50	4.7	26.2	124.9	0.35	
		60	8.1	18.4	80.7	0.26	
	Matrimid 9725	0	0.21 ^a^		28.0		[49]
ZIF-8 250–500 nm		10	0.31 ^a^		29.5		
		20	0.42 ^a^		31.0		
		30	0.7 ^a^		31.5		
	Matrimid 5218	0	8.1	22.4	35.2		[50]
ZIF-8		5	10.1	21.2	39.1		
60 nm		10	13.7	21.6	30.6		
		20	16.6	19.0	35.8		
		30	28.7	17.1	24.9		
ZIF-8	Matrimid	0	10.7		33.9		[51]
Dir. Son.		10	21.9		36.0		
		25	47.0		39.0		
Indir. Son.		10	13.2		31.0		
		25	23.2		31.9		
	Matrimid 5218	0	9.0		35.0		[52]
ZIF-8		15	11.3		35.0		
ZIF-8-ambz		15	10.4		36.5		
		30	10.2		38.0		
	Matrimid 5218	0	9.5	13.6	29.8	0.31	[54]
ZIF-8		10	13.1	20.5		0.26	
95 nm		10 ^b^	15.5	26.7	34.4	0.34	
	P84	0	2.7 ^c^		54.1		[55]
ZIF-8		8	3.2 ^c^		63.5		
30 nm		17	6.3 ^c^		93.6		
		27	11.0 ^c^		92.3		
		31	20.0 ^c^		45.8		
	6FDA-durene	0	468		7		[56]
ZIF-8		5	694		16.5		
50 nm		10	1427		28.7		
		15	1466		11.3		
		20	1463		8.97		
	6FDA-durene	0	469	13.4	15.6	0.91	[57]
ZIF-8		33	1553	11.3	11.1	0.71	
		33 ^d^	23.7	11.8	16.9	0.08	
	6FDA-durene	0	352		16.6		[59]
ZIF-8	T = 200 °C	20	487		17.9		
80 nm	T = 350 °C	0	432		13.8		
		20	857		13.1		
	T = 400 °C	0	541		13.1		
		20	1090		13.0		
	6FDA-durene	0	1468	25.4	22.6		[60]
ZIF-8		3	1593	25.7	21.9		
100–200 nm		5	1695	22.7	20.1		
		7	1774	22.1	19.4		
		10	1882	20.5	19		
		15	1940	18.6	18.1		
		20	2027	17.5	16.9		
		30	2186	17	17.1		
	PEBAX 2533	0	351	35.1	8.3		[61]
ZIF-8		5	305	25.4	6.8		
		10	427	30.5	8.5		
		15	574	30.2	10.4		
		20	854	28.5	9.2		
		25	1082	30.9	8.5		
		30	1176	31.8	8.7		
		35	1287	32.2	9		
	Ultem 1000	0	14 ^e^	30			[63]
ZIF-8		13	26 ^e^	36			
ZIF-7							
	PEBAX 1657	0	72	34	14		[72]
ZIF-7		8	145	68	23		
40–50 nm		22	111	97	30		
		34	41	105	44		
	PEI	0	82.5	3.8	4.4		[73]
ZIF-7		5	64.7	17	12.9		
PSM-ZIF-7 ^g^		5	246	1.3	2.3		
ZIF-11							
	PEBAX 2533	0	232	41.3	8		[77]
ZIF-11		10	212	53	9.7		
500–5000 nm		30	186	47.9	11.4		
		50	233	46.9	11.2		
		70	402	29	12.4		
	6FDA-DAM	0	21.4		32.7		[78]
ZIF-11		10	107		31.3		
200–2000 nm		20	273		31		
		30	76.7		30.4		
ZIF-71							
	PIM-1	0	3265	20.1	10.2		[79]
ZIF-71		10	4271	19.4	11.3		
<1000 nm		20	5942	20	11.9		
		30	8377	18.3	11.2		
	UV-PIM-1	0	1233	29.8	34.1		
UV-ZIF-71		10	1909	29.1	35.5		
<1000 nm		20	2546	27.2	35.3		
		30	3459	26.9	35.6		
ZIF-71	6FDA-Durene	0	805	14.7	17		[76]
30 nm		20	2560	13.8	14.2		
200 nm		20	2744	13.2	13.9		
600 nm		20	1656	13.5	14.7		
ZIF-90							
	6FDA-DAM	0	402		17.5		[80]
ZIF-90		15	808		27.2		
810 nm	Ultem^®^1000	0	1.4		37.9		
ZIF-90		15	2.9		38.9		
	Matrimid	0	7.7		34.9		
ZIF-90		15	12.1		34.8		
	6FDA-DAM ^h^	0	390		24		
		15	720		37		
	6FDA-TP ^i^	0	20	20	37		[81]
ZIF-90		10	26	24	42		
60–105 nm		20	29	22	38		
		40	45	20	36		
		50	63	20	36		

^a^ Permeance (GPU), membrane thickness 40–65 µm; ^b^ ZIF-8 synthesized using the solution collected from freshly-synthesized ZIF-8 dope after centrifugation; ^c^ equimolar CO_2_/CH_4_ mixture; ^d^ membrane surface cross-linked using ethylenediamine vapour; ^e^ Permeance (GPU), membrane thickness ~60 µm; ^f^ Permeance (GPU), membrane thickness 50–100 nm; ^g^ PSM: post-synthetic modification; ^h^ gaseous mixture as feed gas; ^i^ TP: triptycene, 10 atm feed pressure.

**Table 2:** Gas separation performance of UiO-66-based mixed matrix membranes (operating conditions ranging within 1–5 bar, 20–35 °C, unless differently specified).

Filler	Polymer	Loading (wt.%)	P_CO2_ (Barrer)	α _C__O2__/__N2_	α _C__O2__/__CH4_	α _CO__2__/__H2_	Ref.
	PEBAX 1657	0	51.5	42.1			[86]
UiO-66		5	75.0	56.0			
60–80 nm		7.5	90.0	60.0			
		10	96.3	56.6			
		12.5	110.5	40.0			
		15	115.0	27.0			
		20	134.0	21.0			
UiO-66-NH_2_ 60–80 nm		5	71.0	68.0			
		7.5	78.0	76.0			
		10	87.0	79.2			
		12.5	96.0	45.0			
		15	100.0	37.5			
		20	122	26			
	Matrimid 9725 ^a^	0	5.9		31.2		[87]
UiO-66		30	15.0		35.8		
UiO-66-BA		30	17.8		42.9		
UiO-66-ABA		30	13.6		45.1		
UiO-66-NH_2_		30	17.8		37.3		
UiO-66-NH_2_-BA		30	17.4		39.3		
UiO-66-NH_2_-ABA		30	38.0		47.4		
	Matrimid 5218 ^b^	0	8.5	29			[88]
UiO-66 -NH_2_		12	18.5	33			
200 nm		23	24	36			
		40	28	27.5			
UiO-66-NH_2_-PA		12	20.5	32.5			
		23	28	36.5			
		40	31	28			
UiO-66-NH_2_-C10		23	22.5	28			
UiO-66-NH_2_-SA		23	20	30.5			
	PIM-1	0	4770	21.8	16.7	1.76	[89]
UiO-66	as cast	9.1	5940	23.2	16.	1.93	
200 nm		16.6	7610	20.7	14.4	1.67	
		23.1	7610	20.7	14.4	1.67	
		28.6	4940	13.6	11.2	0.66	
UiO-66-(COOH)_2_		9.1	4600	20.9	14.1	2.22	
200 nm		16.6	5190	20.4	13.2	2.19	
		23.1	5300	19.9	12.9	2.22	
		28.6	6090	20.6	15.2	1.63	
UiO-66-NH_2_		9.1	4810	22.2	16.5	1.62	
200 nm		16.6	6340	20.9	14.9	2.03	
		23.1	5070	20.1	14.7	1.58	
		28.6	6310	21.5	13.3	2.10	
	PIM-1	0	8210	21.2	15.7	1.63	
UiO-66	exchanged solvent	16.6	9980	21.6	17	1.23	
200 nm		23.1	9980	21.6	17	1.23	
		28.6	10,900	15.2	13.2	1.74	
UiO-66-(COOH)_2_		16.6	9720	18.9	11.7	2.28	
200 nm		23.1	8770	18.1	11	2.05	
		28.6	9020	22.1	13.5	1.02	
UiO-66-NH_2_		9.1	8740	22	14.7	1.84	
200 nm		16.6	10,700	21.4	13.7	1.88	
		23.1	9570	23.4	13.8	1.43	
		28.6	9030	19.5	13	1.70	
	PIM-1	0	3054	16.1	14.5	1.67	[90]
UiO-66		5	4620	16.2	15.1	1.90	
100–200 nm		10	5210	16.5	13.7	2.04	
		20	6981	13	9.3	2.60	
UiO-66-H		5	2765	22.9	18.2	0.88	
20–30 nm		10	2631	23.5	18.8	0.88	
		20	2606	24.6	20.1	0.89	
		30	1880	18.3	16.1	1.55	
		40	1023	21.4	15.8	1.67	
UiO-66-NH_2_		5	2952	26.9	27.3	1.11	
20–30 nm		10	2869	27.5	28.3	1.09	
		20	2210	23.7	25.1	0.99	
		30	2005	22	23.8	0.99	
		40	1727	24	21.8	0.86	
UiO-66-Br		5	2890	20.1	18.1	1.49	
20–30 nm		10	2846	21.6	17.1	1.25	
		20	2416	19.3	16.3	1.53	
		30	2294	19	17.1	1.57	
		40	1441	23.6	20.8	1.03	
	6FDA-BisP	0	33.9		27.5		[91]
UiO-66		6	56.7		33.6		
50–100 nm		14	83.9		36.2		
		17	108		41.9		
		21	155		24.6		
	6FDA-ODA	0	25.9		20.6		
UiO-66		4	30.1		38		
50–100 nm		8	37.4		51.5		
		17	43.3		57		
		23	72		21.5		
	6FDA-DAM	0	997		29.2		
UiO-66		4	1283		29.6		
50–100 nm		8	1728		32		
		14	1912		30.9		
		21	2358		12.7		
	6FDA-DAM	0	1010 ^c^		29.2		[92]
UiO-66		4	1290 ^c^		29.6		
		8	1730 ^c^		32.1		
		14	1915 ^c^		31.2		
		21	2365 ^c^		12.6		
UiO-66-NH_2_		4	1295 ^c^		29.2		
		8	1300 ^c^		30.3		
		14	1345 ^c^		29.9		
		21	1585 ^c^		20.7		
UiO-66-NH-COCH_3_		4	1081 ^c^		30.3		
		8	1171 ^c^		32.5		
		14	1266 ^c^		33.1		
		21	1417 ^c^		24.1		

^a^ feed pressure = 9 bar; ^b^ feed pressure = 10 bar; ^c^ equimolar CO_2_/CH_4_ gas mixture.

**Table 3:** Gas separation performance of Cu-based MOFs used to prepared mixed matrix membranes (operating conditions ranging within 1–5 bar, 20–35 °C, unless differently specified).

Filler	Polymer	Loading (wt.%)	P_CO2_ (Barrer)	α _C__O2__/__N2_	α _C__O2__/__CH4_	α _CO__2__/__H2_	Ref.
	Matrimid 9725	0	0.21 ^a^		28.0		[49]
Cu_3_(BTC)_2_		10	0.3 ^a^		30.0		
10 μm		20	0.41 ^a^		31.0		
		30	0.64 ^a^		32.5		
Cu_3_(BTC)_2_	PPO	0	68.9	16.1	16.2	0.92	[95]
6 µm		10	87.2	23.8	28.2	0.94	
	PMP	0	76.1	20.5	15.2	7.5	[96]
Cu_3_(BTC)_2_		5	88.3	22.2	17.1	8.1	
100 nm		10	103	23.7	19.2	9.2	
		15	124	25.4	22.7	10.7	
		20	144	28.6	24.3	12.2	
	PEBAX 1657	0	84.2		16.4		[97]
Cu_3_(BTC)_2_		5	91.4		17.7		
		10	102.7		19		
		15	128.8		20.5		
		20	167.3		19.5		
NH_2_-Cu_3_(BTC)_2_		5	93		18.4		
		10	108.8		21		
		15	135.2		23.6		
		20	170.1		26.2		
	Matrimid 5218	0	7.3	30.5	32.8	0.43	[98]
MOP-18		23	9.4	27.6	23.2	0.53	
		33	14	22.9	21.8	0.63	
		44	15.6	26.0	16.4	0.70	
MMIF	Matrimid 5218	0	6.8	26.2	35.9		[6]
50 nm		10	8.1	27.3	36.9		
200 nm		20	8.6	27	34.6		
	Matrimid 5218	0	8.0 ^b^		38.3		
50 nm		10	9.7 ^b^		81		
200 nm		20	10.1 ^b^		88		
	Matrimid 5218	0	7.1 ^c^	32.3			
50 nm		10	8.2 ^c^	38.9			
200 nm		20	11.7 ^c^	58			
CU-BPY-HFS ^d^	Matrimid 5218	0	7.3	33.1	34.7	0.42	[99]
200–300 nm		10	7.81	32.5	31.9	0.46	
		20	9.88	31.9	27.6	0.59	
		30	10.36	33.4	27.4	0.51	
		40	15.06	30.7	25.5	0.56	

^a^ Permeance (GPU); membrane thickness 40–65 µm; ^b^ Equimolar CO_2_-CH_4_ gas mixture; ^c^ Equimolar CO_2_-N_2_ gas mixture; ^d^ Cu-BPY-HFS: Cu–4,4′-bipyridine–hexafluorosilicate.

**Table 4:** Gas separation performance of MIL-based mixed matrix membranes (operating conditions ranging within 1–5 bar, 20–35 °C, unless differently specified).

Filler	Polymer	Loading (wt.%)	P_CO2_ (Barrer)	α _C__O2__/__N2_	α _C__O2__/__CH4_	α _CO__2__/__H2_	Ref.
	Matrimid 5218	0	6.2		28.2		[108]
MIL-53 (Al)		5	6.8		29.6		
123–466 nm		10	7.45		31		
		15	12.43		51.8		
		20	14.52		15.1		
	Matrimid 5218	0	8.4	33.6	39.4	0.33	[109]
MIL-53-as ^a^		37.5	40	95.2	90.1	0.55	
MIL-53-ht ^a^		33.3	26.6	42.9	45.7	0.50	
50–100 nm		37.5	51	28.3	47.0	0.60	
	PMP	0	98.74		8.72		[110]
NH_2_-MIL-53 (Al)		5	107.32		11.85		
110 nm		10	118.74		12.59		
		15	139.56		15.72		
		20	164.78		18.46		
		25	203.44		20.18		
		30	226.37		20.36		
	PVDF	0	0.92	16.3	21.3		[111]
MIL-53		5	1.21	16.3	21.2		
100 nm		10	1.55	16.2	21.0		
NH_2_-MIL-53(Al)		5	1.11	17.3	23.1		
100 nm		10	1.41	19.5	26.0		
	m-PVDF ^b^	0	1.2		27.9		[112]
MIL-53		5	1.75		35.8		
100 nm		10	2.45		39.6		
NH_2_-MIL-53		5	1.69		37.6		
100 nm		10	2.24		43.2		
	6FDA–(DAM)	0	316.6 ^c^		9.76		[113]
MIL-53 (Al)		10	331.9 ^c^		10.19		
190–340 nm		15	354.0 ^c^		11.46		
	6FDA–(DAM)–(HAB) 2:1	0	115.7 ^c^		21.65		
		10	124.2 ^c^		24.62		
		15	134.5 ^c^		26.96		
	6FDA–(DAM)–(HAB) 1:1	0	46.8 ^c^		34.39		
		10	55.3 ^c^		37.15		
		15	63.0 ^c^		40.76		
	6FDA–(DAM)–(HAB) 1:2	0	19.6 ^c^		43.1		
		10	33.2 ^c^		47.13		
		15	42.6 ^c^		48.83		
	6FDA–(DAM)	0	316.2 ^c^		9.77		
NH_2_-MIL-53 (Al)		10	308.9		13.63		
100–200 nm		15	290.7 ^c^		14.77		
		20	299.8 ^c^		8.86		
	6FDA–(DAM)–(HAB) 2:1	0	115.7 ^c^		21.81		
		10	112.1 ^c^		43.63		
		15	105.7 ^c^		36.13		
		20	122.1 ^c^		29.31		
	6FDA–(DAM)–(HAB) 1:1	0	47.4 ^c^		34.54		
		10	43.7 ^c^		77.72		
		15	44.6 ^c^		64.54		
		20	54.7 ^c^		35.68		
	6FDA–(DAM)–(HAB) 1:2	0	24.6 ^c^		53.86		
		10	20.0 ^c^		86.81		
		15	21.9 ^c^		96.36		
		20	31.9 ^c^		55.9		
	PDMS	0	30	23.3	27.0	0.22	[114]
P-MIL-53		5	33.3	24.5	28.8		
500 nm		10	36.0	25.8	30.5	0.24	
		15	40.3	28.1	32.1		
		20	42.3	27.5	28.4		
	Matrimid 5218 ^d^	0	4.44	34	35		[116]
MIL-101(Cr)		10	6.95	52	56		
~1000 nm		15	5.7	44	47		
		20	5.85	42	37		
		30	7.99	47	44		
	Matrimid 5218 ^f^	0	7.33		34.9		[117]
MIL-101(Cr)		10	12.01		52.21		
	Matrimid/PVDF ^f^	0	9.42		42.81		
MIL-101(Cr)		10	14.87		62		
	PPO-PEG ^c,e^	0	657		18.42		[118]
MIL-53(Al)-PC		5	684		25.51		
200–250 nm		10	723.6		29.23		
		15	763		35.78		
		20	789		40.39		
		25	1266		31.53		
MIL-101(Cr)-PC	PPO-PEG ^c,e^	0	657		19.26		
50–100 nm		5	771		22.93		
		10	874		26.61		
		15	952		30.46		
		20	1056		34.66		
		25	1896		29.24		
	PSF	0	5		23		[119]
MIL-101		8	8		21		
110–400 nm		16	8.9		24		
		24	18.1		28		
ZIF-8		0	5		23		
75–100 nm		8	10		35		
		16	14		22		
		24	24		24		
MIL-101/ZIF-8		0	4.7		23		
		8	10.6		36		
		16	14.2		40		
		24	24		26		
		35	29.6		24		

^a^ as = as synthesized, “ht” = high temperature treated (300 °C); ^b^ m-PVDF = modified poly(vinylidene fluoride); ^c^ mixed gas conditions; ^d^ feed side pressure = 10 bar; ^e^ PPO-PEG = polyphenylene oxide-polyethylene glycol; ^f^ feed pressure = 7 bar; ^g^ Permeance (GPU); ^h^ MSxPy: MOFs Scaffold.

**Table 5:** Gas separation performance of different MOFs (Fe(BTC), KAUST-7, Mg_2_(dobdc)) used to prepared mixed matrix membranes (operating conditions ranging within 1–5 bar, 20–35 °C, unless differently specified).

Filler	Polymer	Loading (wt.%)	P_CO2_ (Barrer)	α _C__O2__/__N2_	α _C__O2__/__CH4_	α _CO__2__/__H2_	Ref.
	Matrimid 5218	0	9		25		[121]
Fe(BTC)		10	9.5		27.5		
		20	10.8		28		
		30	13.1		29.5		
	Matrimid 5218 ^a^	0	14.6	4.4			[122]
Fe(BTC)		10	84.9	43.5			
10–20 um		20	91.2	15.4			
		30	217.9	23.1			
	Pebax 1657	0	70.67		18.4		[123]
Fe(BTC)		5	80.79		19.3		
		10	82.32		19.4		
		15	89.63		20.8		
		20	98.32		22.2		
		25	148.44		21.9		
		30	402.69		21.5		
		40	425.5		12.3		
		0	60.35 ^b^		16.9		
		10	70.11 ^b^		17.6		
		20	85.28 ^b^		19.3		
		30	329.7 ^b^		20.5		
		40	345.4 ^b^		13.1		
	6FDA Durene	0	759.7 ^b^		34.7		[125]
KAUST-7		11	895.7 ^b^		36.2		
80 nm		22	966.9 ^b^		37.0		
		33	1038.1 ^b^		37.6		
	PDMS	0	3100.0	9.5			[126]
Mg_2_(dobdc)		20	2100.0	12			
100 nm	XLPEO	0	380.0	22			
		10	250.0	25			
	6FDA-TMPDA	0	650.0	14			
		10	850.0	23			

^a^ Temperature = 80 °C; ^b^ Mixed gas CO_2_/CH_4_ 10/90.

**Table 6:** Gas separation performance of porous organic frameworks (POFs)-based mixed matrix membranes (operating conditions ranging within 1–5 bar, 20–35 °C, unless differently specified).

Filler	Polymer	Loading (wt.%)	P_CO2_ (Barrer)	α _C__O2__/__N2_	α _C__O2__/__CH4_	α _CO__2__/__H2_	Ref.
	PTMSP – 0 d	0	20,000	8.7			[135]
PAF-1		10	25,000	8.1			
	PTMSP – 240 d	0	12,400	9.8			
PAF-1		10	23,200	9.6			
	PIM-1 – 0 d	0	4000	15			
PAF-1		10	15,000	12			
	PIM-1 – 240 d	0	1700	19			
PAF-1		10	15,000	19			
	PMP– 0 d	0	6500	10.5			
PAF-1		10	11,500	9.4			
	PMP – 240 d	0	3500	11			
PAF-1		10	10,500	9.4			
	PTMSP	0	30,000	5.6			[136]
PAF-11		1	38,000	5.9			
		5	37,000	5.8			
		10	34,000	5.6			
	510 hours	1	20,000	7			
		5	19,500	6.8			
		10	23,500	6.3			
	PTMSP	0	30,000	5.9	2.3		[137]
PAF-1		10	35,500	5.7	2.3		
PAF-1-NH_2_		10	43,000	5.9	2.2		
PAF-1-SO_3_H		10	32,500	5.7	2.3		
PAF-1-C_60_		10	33,000	5	2.1		
PAF-1-Li_6_C_60_		10	55,000	5.4	2		
	Aged	0	8000	8.8	5.3		
PAF-1		10	28,000	7.4	3.1		
PAF-1-NH_2_		10	29,000	7.5	3.6		
PAF-1-SO_3_H		10	23,500	6	2.6		
PAF-1-C_60_		10	15,000	8.3	5		
PAF-1-Li_6_C_60_		10	50,600	9	3.9		
	PIM-1 - CH_2_Cl_2_	0	2258	24			[138]
HCP		5.7	4690	17.6			
		16.67	5103	13.1			
		21.3	6331	14.1			
	150 d	0	1109	4.2			
HCP		5.7	3616	19.7			
		21.3	5060	16			
	PIM-1 - CHCl_3_	0	2660	22.3			
HCP		4.6	4313	19.8			
		9.1	4700	19.3			
		16.67	10,040	17.1			
	150 d	0	1225	21.5			
HCP		4.6	1857	22.4			
		9.1	2043	22.2			
		16.67	4165	21.8			

**Table 7:** Gas separation performance of zeolites-based mixed matrix membranes (operating conditions ranging within 1–5 bar, 20–35 °C, unless differently specified).

Filler	Polymer	Loading (wt.%)	P_CO2_ (Barrer)	α _C__O2__/__N2_	α _C__O2__/__CH4_	α _CO__2__/__H2_	Ref.
	PEBAX 1675	0	120		20.3		[143]
ZSM-5		5	230		21		
		10	191		32.5		
		15	170		33.9		
	Matrimid 5218	0	5.1 ^a^		14.8		[144]
ZSM-5		6	6.6 ^a^		15.6		
		15	11.1 ^a^		7.2		
		24	14.5 ^a^		4.8		
		30	21 ^a^		3.6		
	PEBAX 1675	0	81.4	41			[145]
13X		10	104	39			
		15	114	47			
	PEBAX 1675	0	55.8	39.9	18.0		[147]
4A		5	71.4	51.0	32.5		
		10	97	53.9	26.2		
		20	113.7	39.2	17.5		
		30	155.8	13.0	7.9		
	PEBAX 1675	0	110	54	16	8.99	[148]
SAPO-34		9	100	53	16.5	8.29	
		23	130	56	21.9	6.58	
		33	250	55.7	16.4	8.96	
		50	340	55.5	16.5	8.40	
	PDMS	0	4796		3.0	4.21	[150]
4A		10	4226		2.7	1.55	
		20	3691		2.6	0.61	
		30	3323		2.9	0.40	
		40	2972		2.8	0.30	
		50	2886		2.9	0.27	
	PVAc	0	2.74	28	53		[151]
Ferrierite		20	3.93	61	54		
		40	3.93	82	57		
4A		20	2.55	52			
		40	2.73	74			
5A		20	2.77	46			
		40	1.70	33			
Silicalite-1		20	3.38	42			
		40	3.52	50			
	PTMSP	0	17430	0.9			[152]
Zeolite A		5	13029	9.7			
		20	11403	76.4			
ITQ-29		5	16501	4.4			
		20	14546	1.1			
	PSF	0	4.9		18.5		[154]
4A		20	5		12.5		
		25	6.9		7.6		
		30	7		2		
		35	7.12		1.44		
treated 4A		20	4.75		23.5		
		25	4.73		28		
		30	4.7		31		
		35	3.7		29		
	Matrimid	0	10.2		33.6		[155]
5A		10	26.7		31.3		
		20	31		30.8		
5A-Mg(OH)2		10	19.6		35.4		
		20	22.4		36.4		
	Cellulose Acetate	0	2.2	26			[156]
Na-Y		5	2.5	22.5			
		10	2.6	22			
		15	3.4	21			
		20	4.95	22.5			
		25	3.5	15			
Na-Y-NH2		5	3.2	25			
		10	3.5	23			
		15	3.65	22			
		20	4.1	26			
		25	4.3	17			

^a^ Permeance (GPU); membrane thickness 3–5 µm.

**Table 8:** Gas separation performance of mixed matrix membranes containing MOFs nanosheets (operating conditions ranging within 1–5 bar, 20–35 °C, mixed gas conditions unless differently specified).

Filler	Polymer	Loading (wt.%)	P_CO2_ (Barrer)	α _C__O2__/__N2_	α _C__O2__/__CH4_	α _H__2__/__CO2_	Ref.
	Cellulose Acetate	0	7.55		29.61		[163]
AMH-3		2	9.65		29.24		
		4	10.36		30.03		
		6	11.59		29.71		
	Ultem	0 ^a^	2.22		20.2	2.88	[164]
NUS-2		10 ^a^	3.75		25	3.39	
		20 ^a^	4.92		22.4	4.61	
		30 ^a^	8.70		12.7	1.89	
NUS-3		10 ^a^	5.89		22.7	2.46	
		20 ^a^	15		28.3	2.23	
		30 ^a^	8.11		10.7	2.45	
	Matrimid 5218	0	5.78		59.8		[165]
ns-CuBDC ^b^		1.7	5.38		61.6		
		3.7	9.91		59.5		
		4.3	4.74		63.5		
		8.2	4.09		78.7		
b-CuBDC ^b^		7.9	5.21		45		
nc-CuBDC ^b^		8.3	5.03		49.4		
	Matrimid 5218	0	7.2 ^c^	23.7			[166]
CuBDC		4	6.4 ^c^	42.0			
		8	4.0 ^c^	48.1			
		0	15.2		25.3		
		12	6.6		40.3		
	PIM-1	0	1750 ^d^		4.4		[167]
CuBDC-ns		2	500 ^d^		10.2		
		5	490 ^d^		12.9		
		10	400 ^d^		16.0		
		15	490 ^d^		11.7		
		0	161 ^d^		12.2		
		10	196 ^d^		10.8		
		10	407 ^d^		15.5		
	PIM-1	0	3100		17		[168]
CuBDC-ns		2	2030		24		
		4	2300		22		
	6FDA-DAM	0	590		30		
CuBDC-ns		2	570		37		
		4	430		43		
	PBI	0	3.62			9.3	[169]
ns-Cu_2_(ndc)_2_(dabco) ^b^		10	4.86			18.7	
		20	6.15			22.8	
		30	11.9			12.3	
		50	66.4			4.8	
bc-Cu_2_(ndc)_2_(dabco) ^b^		20	5.18			12.6	
nc-Cu_2_(ndc)_2_(dabco) ^b^		20	5.29			17.6	

^a^ Operating pressure of 2 bar, ^b^ ns = nanosheets; bc = bulk crystals; nc = nano crystals; ^c^ single gas tests; ^d^ permeance (GPU).
